# Low expression of m6A reader YTHDC1 promotes progression of ovarian cancer via PIK3R1/STAT3/GANAB axis

**DOI:** 10.7150/ijbs.81595

**Published:** 2023-08-28

**Authors:** Xiaogang Wang, Qianyu Chen, Ziqian Bing, Shizhen Zhou, Zhen Xu, Yayi Hou, Yue Zhao, Shuli Zhao, Tingting Wang

**Affiliations:** 1 The State Key Laboratory of Pharmaceutical Biotechnology, Division of Immunology, Medical School, Nanjing University, Nanjing, China.; 2 Jiangsu Key Laboratory of Molecular Medicine, Division of Immunology, Medical School, Nanjing University, Nanjing, China.; 3 General Clinical Research Center, Nanjing First Hospital, Nanjing Medical University, Nanjing, China.

**Keywords:** m6A reader, YTHDC1, PIK3R1, RNA stability, ovarian cancer

## Abstract

**Background:** N6-Methyladenosine (m6A) is considered to be the most prevalent and abundant internal modification observed in mRNA between viruses and mammals. As a reversible epigenetic modification, m6A controls gene expression in diverse physiological and pathological processes. Accumulating evidence in recent years reveals that aberrant expression of m6A reader proteins may have tumor-suppressing or carcinogenic functions. However, the biological role and mechanism of m6A reader YTH Domain Containing 1 (YTHDC1) in ovarian cancer progression remain inadequately understood.

**Methods:** Quantitative RT-PCR, immunohistochemistry, Western blot, and bioinformatics analyses were undertaken for studying the YTHDC1 expression in ovarian cancer. *In vitro* and *in vivo* models were used to examine the role of YTHDC1. RNA sequencing, RNA immunoprecipitation sequencing, m6A-modified RNA immunoprecipitation, actinomycin-D assay, chromatin immunoprecipitation, and Western blot were used in the investigation the regulatory mechanisms among YTHDC1, Signal Transducer and Activator of Transcription 3 (STAT3), Phosphoinositide-3-Kinase Regulatory Subunit 1 (PIK3R1), and Glucosidase II Alpha Subunit (GANAB).

**Results:** Here, we found that YTHDC1 expression is decreased in ovarian cancer. Overexpression of YTHDC1 inhibited ovarian cancer development both *in vivo* and *in vitro*. Mechanistically, PIK3R1 was identified to be the direct target for YTHDC1. YTHDC1 enhanced PIK3R1 stability in an m6A-dependent manner, which subsequently inhibited GANAB expression in the N-glycan biosynthesis via the STAT3 signaling.

**Conclusions:** Our findings unveil YTHDC1 as a tumor suppressor in the progression of ovarian cancer and as a potential prognostic biomarker that could serve as a target in ovarian cancer treatment.

## Introduction

Ovarian cancer is one of the top three most frequent gynecologic malignancies in the world. It continues to have the maximum fatality rate because of its high recurrence rate and poor prognosis 1, 2. Moreover, ovarian cancer is typically diagnosed at a late stage and has no efficient screening strategy 3. Despite advances in treatments with surgery and chemotherapy for ovarian cancer patients, the prognosis has only slightly improved 4, 5. Therefore, it is anticipated that investigations on the putative molecular basis for the emergence and progression of ovarian cancer will serve as a theoretical foundation for the development of novel therapeutic approaches.

Recently, surging evidence has elucidated a crucial function for epigenetic modification in the growth of tumors. m6A is recognized as the most common, abundant, and conserved internal modification of mRNA and non-coding RNAs (ncRNAs) in most eukaryotic species 6-8. As a dynamic and reversible process, it plays an important role in almost all essential bioprocesses, which include the differentiation of stem cells, the development of tissue, and the progression of cancer 9-12. The catalysis of the m6A modification happens with the help of a protein complex that consists of m6A methyltransferases (METTL3, METTL14, and WTAP; also called writers). The m6A modification is removed by two demethylases FTO and ALKBH5, also called erasers 13-15. However, the fate and function of m6A-modified RNA are mainly controlled by the readers (YTH domain proteins, namely, YTHDC1, YTHDC2, and YTHDF1-3), which recognize and bind to the m6A motif and regulate RNA splicing, translation, nuclear export, and RNA stability 16. Notably, the m6A modification exerts either a carcinogenic or a tumor-suppressing effect in different scenarios due to different readers 17, 18. Nevertheless, there is still not fully elucidated about the biological function of m6A and its potential regulatory mechanisms in ovarian cancer.

In this study, we aimed to unravel the functions of m6A modifications (especially the reader YTHDC1) in regulating ovarian cancer progression. Contemporary research has shown that YTHDC1 maintains the stability and nuclear export of m6A-modified RNAs by binding m6A sites to regulate several human cancers 19-21. Herein, we first observed that YTHDC1 underwent significant downregulation in ovarian cancer. Our results showed that the overexpression of YTHDC1 could inhibit the progression of ovarian cancer both *in vivo* and *in vitro*. Mechanistically, we found that YTHDC1 binds and stabilizes PIK3R1 in an m6A-dependent manner and further inhibits GANAB-mediated N-glycan biosynthesis through the STAT3 signaling pathway. Therefore, we concluded that YTHDC1 may play the important role of a tumor repressor in ovarian cancer progression, which sheds light on the targeted therapy for ovarian cancer.

## Materials and methods

### Tumor samples

Ovarian cancer and benign ovarian tissues used in this study were all obtained from Nanjing First Hospital as paraffin-embedded (FFPE) samples fixed with formalin and as samples of fresh tissue. Expert pathologists assessed each of these samples and validated the samples' diagnoses as disease samples. Before beginning our experiment, we received informed consent in writing from each participant. The Ethics Committee of Nanjing First Hospital gave their approval for this investigation using patient samples.

### Cell culture

HEK293T, SKOV3, and OVCAR3 cells were obtained from American Type Culture Collection (ATCC). The A2780 cells were obtained from Biobw (Beijing, China). SKOV3 cells were cultivated in McCoy's 5A (Gibco, USA) medium whereas A2780, OVCAR3, and HEK293T cells were cultured in DMEM (Gibco, USA) medium. 10% Fetal Bovine Serum (FBS) (Gibco, USA) and 1% (w/v) Penicillin-Streptomycin (Gibco, USA) were added to the medium as supplements. The cells above were kept at 37 ℃ in a cell culture incubator with 5% CO_2_.

### Plasmid construction, small interfering RNA (siRNA) transfection and lentiviral infection

The lentivirus plasmid overexpressing YTHDC1 and PIK3R1 (OE-YTHDC1 and OE-PIK3R1) were synthesized by Syngentech (Beijing, China). The human PIK3R1-CDS sequence or GANAB-CDS sequence was synthesized by Tsingke Biotechnology (Beijing, China), which was further subcloned into a pcDNA3.1 vector obtained from Addgene (Cambridge, MA). Complementary oligonucleotides (sense and antisense) encoding shRNA which target YTHDC1 (shYTHDC1) and GANAB (shGANAB) were synthesized by Tsingke Biotechnology (Beijing, China) and cloned into the pLKO.1 vector (Addgene, Cambridge, MA) to construct the shRNA plasmid used for interference studies mediated by lentivirus. In addition, designing and synthesis of siRNAs targeting YTHDC1 (si-YTHDC1), PIK3R1 (si-PIK3R1), STAT3 (si-STAT3), METTL3 (si-METTL3), METTL14 (si-METTL14), and WTAP (si-WTAP) were done by RiboBio (Guangzhou, China). Transfection of the cells with siRNAs was performed using Lipofectamine RNAiMAX (Invitrogen, CA, USA) and transfection with plasmids using Lipofectamine 2000 (Invitrogen, CA, USA). Lentiviral vectors were co-transfected with packaging vectors psPAX2 and pMD2.G (Addgene, Cambridge, MA) using Lipofectamine 2000 (Invitrogen, CA, USA) to produce lentiviruses. At 48 hours after transfection, infectious lentivirus particles were collected and transduced into the target cells. Infected cells were selected 48 hours later using 1 μg/ml puromycin (Beyotime Biotechnology, China). The above sequences are provided in Table S1.

### *In vivo* tumorigenesis assay

5-6 weeks old BALB/c male nude mice were procured from the Nanjing Biomedical Research Institute of Nanjing University and kept in an SPF facility. For tumorigenesis assay: 1 SKOV3 and OVCAR3 cells were stably infected with OE-YTHDC1 or lentiviral vectors; 2 SKOV3 cells were stably infected with shYTHDC1, shYTHDC1 with OE-PIK3R1, or lentiviral vectors; 3 SKOV3 cells were stably infected with shYTHDC1, shGANAB, shGANAB with shYTHDC1, or lentiviral vectors. The different infected ovarian cancer cells (5 × 10^6^) were injected subcutaneously into the right axilla of five mice per group. Mice were euthanized after 4-6 weeks, and the tumors were weighed and imaged.

### RNA extraction and quantitative real-time PCR (qRT-PCR)

TRIzol reagent (Vazyme, Nanjing, China) was used to extract the total RNA from cell samples. Using the Cytoplasmic & Nuclear RNA Purification Kit (Norgen Biotek, Torold, ON, Canada) in accordance with the manufacturer's instructions, cytoplasmic and nuclear RNA was isolated. With the use of HiScript® III RT SuperMix (+gDNA wiper), RNA was reverse transcribed into cDNA (Vazyme, Nanjing, China) for qRT-PCR. The ABI StepOne Plus with PowerUp™ SYBR® Green (Applied Biosystems, USA) was used for qRT-PCR analyses. The internal control for normalization was β-actin. Table S2 includes a list of the qRT-PCR primers.

### Western blot assay and antibodies

The OVCAR3, SKOV3, and A2780 cells with various treatments were double-washed with cold PBS and lysed on ice using RIPA (radioimmunoprecipitation assay) containing 1% PMSF (Solarbio, Beijing, China). Using the nuclear protein and cytoplasmic protein extraction kit (Beyotime, Shanghai, China) in accordance with the manufacturer's instructions, cytoplasmic and nuclear protein was isolated. Protein concentration was assessed using a BCA protein assay kit (Beyotime, Shanghai, China) after the lysate was recovered by centrifugation at 12000 g for 10 min. Then, 5×loading buffer was added to the lysate and heated for 10 min at 100 °C. Using 10% SDS-PAGE, the proteins were separated and transferred to 0.45 μm polyvinylidene fluoride (PVDF) membranes (Millipore, USA); blocked for two hours at room temperature in using a 5% skim milk solution followed by incubation with specific primary antibodies at 4 °C overnight. Secondary antibodies conjugated with HRP were incubated at room temperature for one hour. The following antibodies were used: anti-GAPDH (1:1000, #2118, CST); anti-YTHDC1 (1:500, ab122340, Abcam); PIK3R1 (1:1000, #5295, CST); METTL3 (1:1000, ab195352, Abcam); METTL14 (1:1000, #51104, CST); GANAB (1:2000, ab176349, Abcam); STAT3 (1:2000, #4904, CST); p-STAT3 (1:2000, #9145, CST); anti-β-actin (1:1000, #8457, CST); anti-Histone-H3 (1:2000, 17168-1-AP, Proteintech); anti-α-Tubulin (1:20000, 66031-1-Ig, Proteintech).

### Cell growth and proliferation assays

The viability of the cells was assessed using a cell counting kit-8 assay (CCK8). In 96-well plates, seven independent replicates of the various treated OVCAR3, A2780, and SKOV3 cells were incubated. 10% CCK8 (DOJINDO, Japan) was added to the treated cells and incubated for two hours at 37℃ to evaluate the viability of the cells. Using a microplate reader set at 450 and 630 nm, the absorbance of each well was determined.

5-Ethynyl-2'-deoxyuridine (EdU) assays were operated following the manufacturer's instructions using the EdU Cell Proliferation Assay Kit (RiboBio, Guangzhou, China).

For CFSE (CFDA-SE) assay, SKOV3 and A2780 cells were labeled with CellTrace™ CFSE Cell Proliferation Kit (Invitrogen, CA, USA) according to the manufacturer's protocol. Before their analysis using a BD Accuri C6 Flow cytometer (BD Biosciences, USA) the cells were treated with si-YTHDC1 for 36 h. For colony formation assay, in six-well plates, the 1 × 10^3^ OVCAR3, A2780, and or SKOV3 infected cells were seeded for 10-12 days. Then, they were stained for 20 min with 0.1% crystal violet (Solarbio, Beijing, China) and washed thoroughly with PBS.

### Transwell assay

The invasion and migration capacities of the cells were evaluated using 24-well Transwell chambers (Corning, USA). Briefly, in 0.2 mL of serum-free medium, the A2780, SKOV3, and OVCAR3 infected cells were seeded in the upper chambers. The chambers were coated with Matrigel (Corning, USA) for invasion and without the Matrigel coating for migration assays; 0.6 mL of medium with 10% FBS was added into the lower chambers. Cells were fixed for 20 min with 4% paraformaldehyde after cultivation 24-48 h, stained for 30 min with crystal violet (Solarbio, Beijing, China), and imaged.

### Immunohistochemistry (IHC)

Firstly, we dewaxed and rehydrated paraffin-embedded mouse and human sections. Then, the antigen was extracted with 10 mM citrate solution, and blocking using PBS supplemented with 5% bovine serum albumin (BSA). The sections were incubated with primary antibodies at 4 °C for a night, followed by an hour at room temperature with HRP-conjugated secondary antibody. Following the manufacturer's instructions, the DAB Substrate Kit was used to identify the immune complexes. The following antibodies were used: anti-YTHDC1 (1:500, ab122340, Abcam); anti-Ki-67 (1:1000, #9449, CST); anti-PIK3R1 (1:100, 60225-1-Ig, Proteintech); anti-caspase-3 (1:1000, #9662, CST); anti-GANAB (1:100, ab176349, Abcam).

### RNA sequencing (RNA-seq)

We carried out RNA-seq on the Illumina Hiseq sequencing platform provided by OE Biotech Co., Ltd (Shanghai, China). Briefly, total RNA from SKOV3 and A2780 cells with YTHDC1 knockdown was isolated using a TRIzol reagent. After purification using the PolyTtract mRNA Isolation System, cDNA libraries were generated using the poly(A) RNA. HISAT2 software was used to map the sequence reads to the human genome version hg19. The following analyses employ the average gene expression values from three independent investigations.

### RNA immunoprecipitation and high-throughput sequencing (RIP-seq)

The Millipore Magna RIP Kit (Millipore, Darmstadt, Germany) was used to conduct the RIP assay following the directions provided by the manufacturer. Briefly, the RIP lysis buffer, which contains protease and RNase inhibitor, was used to lyse the SKOV3 and A2780 cells. The cell lysate supernatant was then subjected to overnight incubation at 4 °C with anti-YTHDC1 (ab264375, Abcam) antibody-conjugated beads. The binding complexes underwent thorough washing, elution, purification to extract RNAs, and qRT-PCR analysis. The collection of RNAs was normalized to IgG. Additionally, LC-BIO (Hangzhou, China) carried out the high-throughput sequencing.

### m6A-modified RNA immunoprecipitation (MeRIP) analyzes

The m6A modification levels of YTHDC1 targeted mRNA were performed by a Magna MeRIP™ m6A kit (Millipore, Darmstadt, Germany) following the directions provided by the manufacturer. In brief, total RNA (> 300µg) was extracted from the SKOV3 and A2780 cells. One-tenth of the total RNA was saved as input; the remaining RNAs were immunoprecipitated using m6A antibody-conjugated magnetic beads. The binding complexes were washed with immunoprecipitation buffer, then eluted with elution buffer, purified to obtain RNAs, and analyzed by qRT-PCR. The enrichment of RNAs was normalized to IgG.

### Chromatin immunoprecipitation (ChIP)

To evaluate binding ability of STAT3 to GANAB promoter, a ChIP experiment was carried out following the instructions of a ChIP kit (BersinBio, Guangzhou, China). Briefly, transfected cells were washed twice with PBS, then crosslinked, lysed and sonicated to obtain appropriate fragments. The sonicated solution of chromatin was immunoprecipitated with anti-STAT3 at 4 °C overnight. To obtain immunoprecipitated DNA, the binding complexes were washed, eluted, purified, and then evaluated by qRT-PCR. Table S3 lists the primers used for the ChIP assay.

### Lectin fluorescence histochemistry

As the previously reported protocol, FITC-labeled lectins were used to identify the specific sugar structure present in the cells 22. Briefly, transfected SKOV3 and A2780 cells were subjected to fixation and permeabilization, followed by blocking with 4% BSA for 30 min in PBS at 37 °C. Next, FITC-labeled lectin (PHA-E and PHA-E+L) diluted at a final concentration of 15-20 μg/mL was used to incubate the cells for 3 h at room temperature in the darkness. Nuclei were counterstained using DAPI. An FV3000 confocal fluorescence microscope was used to capture the images.

### Statistical analyses

The mean ± SEM was used to express the experimental results. GraphPad Prism 8.0 software was used to conduct a one-way analysis of variance (ANOVA) and t-tests on all the data in this work. The results were deemed statistically significant based on the criterion of *P*-value < 0.05 (*P< 0.05, **P < 0.01, and ***P < 0.001).

## Results

### YTHDC1 is poorly expressed in ovarian cancer

**Figure 1:**
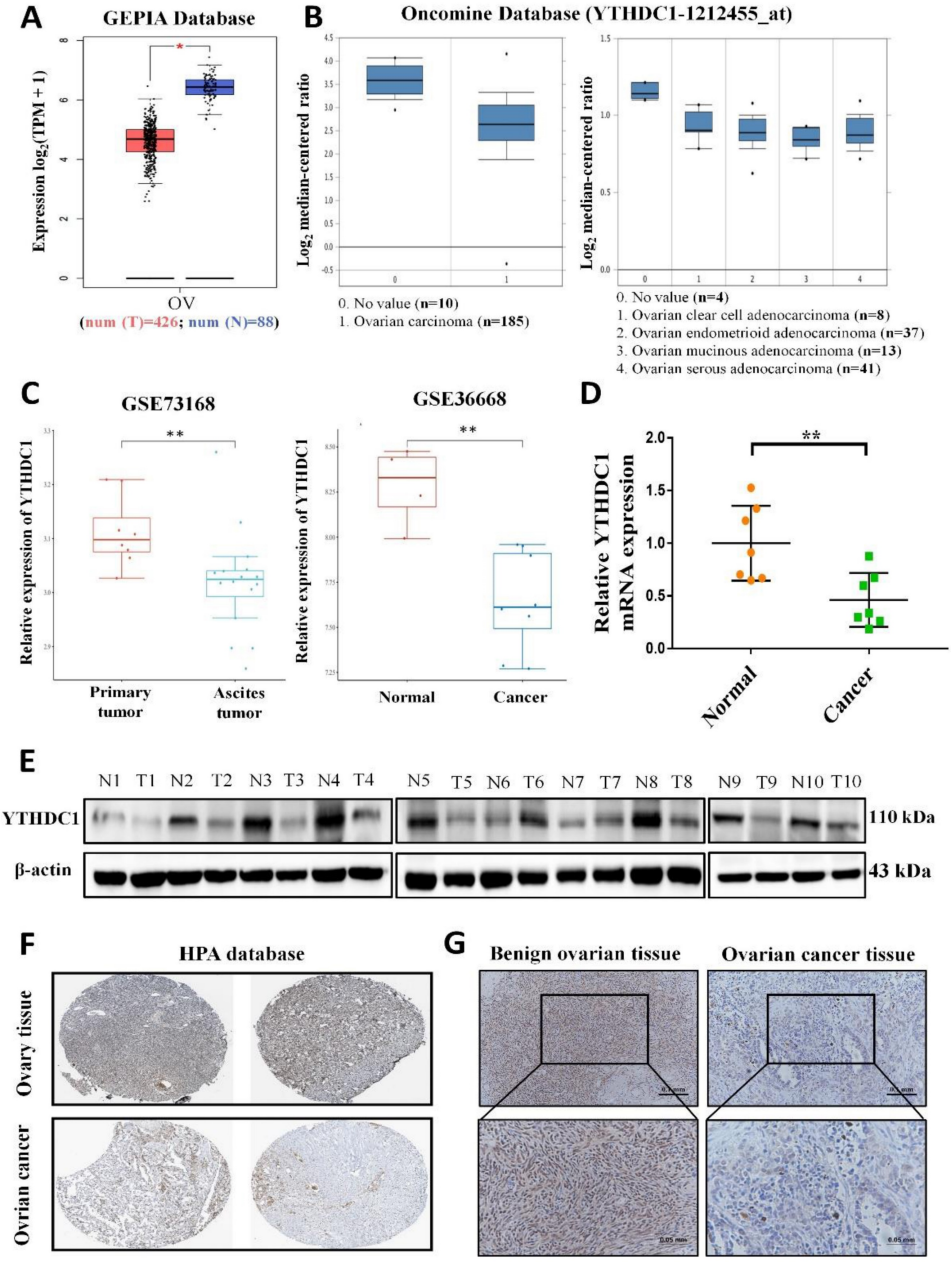
Lowered YTHDC1 expression level in ovarian cancer. (A-C) Relative YTHDC1 RNA level in ovarian cancer tissues and normal tissues in GEPIA (A) and Oncomine (B) data base and GEO datasets (C). (D and E) The YTHDC1 expression level in ovarian cancer tissues and benign ovarian tissues measured using qRT-PCR (D) and western blot (E). (F) Immunohistochemical images of YTHDC1 expression in ovarian cancer tissues and ovary tissues in the HPA database. (G) Illustrative immunohistochemistry images of YTHDC1 expression in cancerous and benign ovarian tissue. Scale bar, 100μm or 50μm.

To examine the potential role of the m6A reader YTHDC1 in the progression of ovarian cancer, we firstly analyzed YTHDC1 expression level in ovarian cancer. Our analysis using the GEPIA and Oncomine database revealed that the expression of YTHDC1 was remarkably lower among ovarian cancer tissues as compared to normal tissues of the ovary (Figure 1A-B). Moreover, the GEO datasets also confirmed that there was significant downregulation of the YTHDC1 mRNA in the case of ovarian cancer (GSE36668, Figure 1C right) and low expression in ascites ovarian cancer compared to primary ovarian cancer (GSE73168, Figure 1C left). Consistent with these findings, the RNA and protein (8/10, 80%) levels of YTHDC1 in ovarian cancer tissues were found to be remarkably lower compared to benign ovarian tissues (Figure 1D-E). Additionally, we observed by IHC staining that there was a significant decrease of YTHDC1 protein level in ovarian cancer tissues as compared to benign ovarian tissues of humans (Figure 1F-G and Figure S1). The above results suggest that the m6A reader YTHDC1 is lowly expressed in ovarian cancer.

### Low expression of YTHDC1 accelerates the progression of ovarian cancer

**Figure 2:**
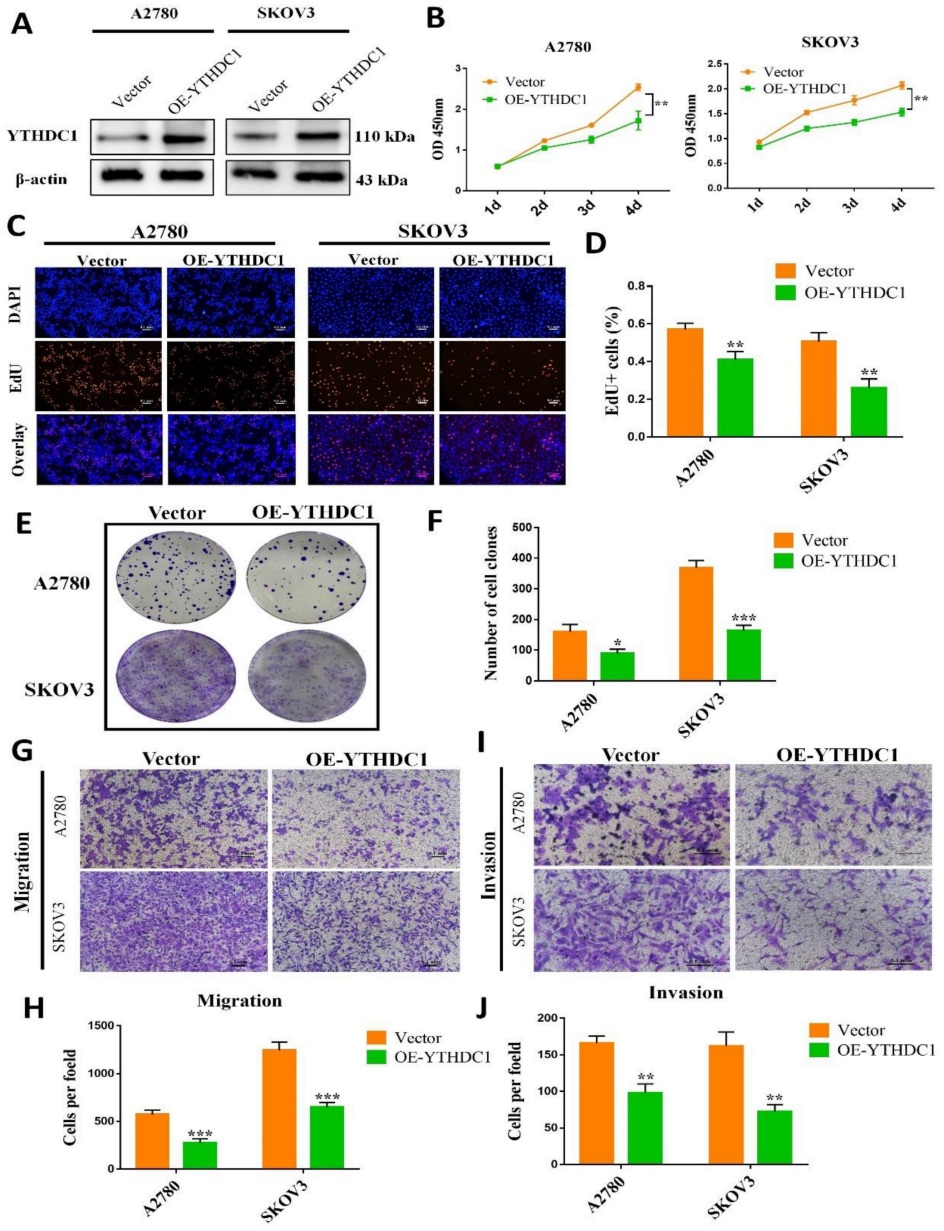
Overexpression of YTHDC1 inhibits the development of ovarian cancer. (A) YTHDC1 protein level in SKOV3 and A2780 cells with YTHDC1 overexpression evaluated by western blot. (B) CCK-8 assays for the determination of cell proliferation post overexpression of YTHDC1 in SKOV3 and A2780 cells. (C and D) EdU assays to detect the rate of proliferation of SKOV3 and A2780 cells. Scale bar, 100 μm. (E and F) Colony formation assays of SKOV3 and A2780 cells to detect proliferation. (G-J) Transwell assays to evaluate cell migration (G and H) and invasion (I and J) using infected SKOV3 and A2780 cells. Data are presented as means ± SD, *P < 0.05, ** *P* <0.01, ***P < 0.001.

*In vitro* and* in vivo* evaluation of the function of YTHDC1 in ovarian cancer, we first established stable ovarian cancer cell lines of YTHDC1-overexpressing (OE-YTHDC1) (Figure 2A). EdU and CCK-8 assays indicated that the growth of ovarian cancer cells was remarkably suppressed with YTHDC1 upregulation (Figure 2B-D and Figure S2A).

Furthermore, the clonogenicity was found to be inhibited after overexpression of YTHDC1 as suggested by the colony-formation assays (Figure 2E-F and Figure S2B-C). Additionally, overexpression of YTHDC1 was found to impair the migration (Figure 2G-H and Figure S2D-E) and invasion (Figure 2I-J and Figure S2D-E) capacity of A2780, SKOV3, and OVCAR3 cells as revealed by the transwell assays. However, contrary to the above results, the knockdown of YTHDC1 significantly promoted of ovarian cancer cell proliferation, migration and invasion Figure S3.

**Figure 3:**
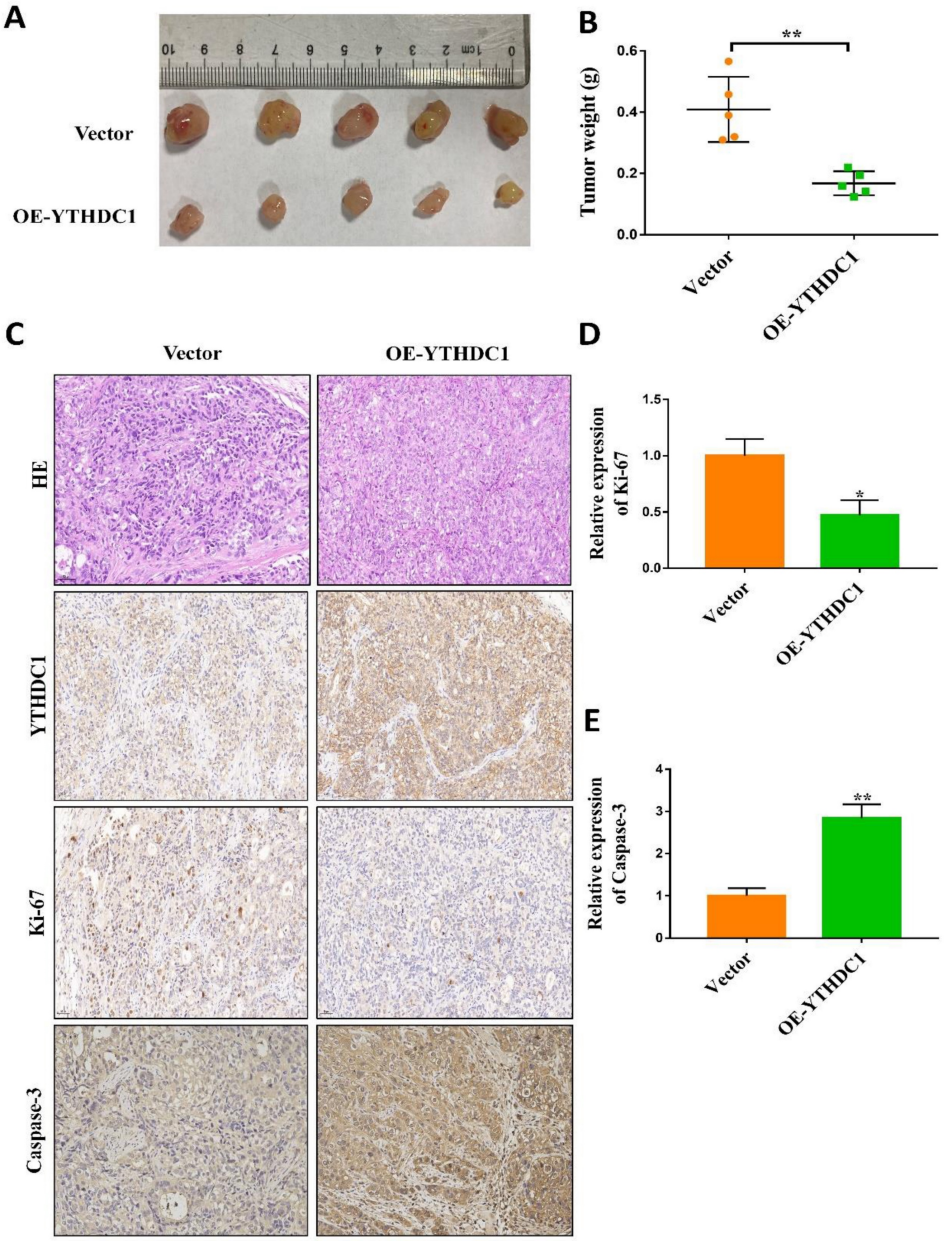
YTHDC1 overexpression suppresses tumorigenesis *in vivo*. (A and B) SKOV3 cells with empty vector or OE-YTHDC1 injected subcutaneously into five nude mice. The tumors were extracted, photographed (A) after 45 days transplantation and weighed (B). (C-E) Representative immunohistochemistry and quantification data of YTHDC1, caspase-3, and Ki-67 by positive staining in tumors. Scale bar, 50μm. Data presented as means ± SD, *P < 0.05, **P < 0.01.

To study the oncogenic property of YTHDC1 in *in vivo* ovarian cancer models, control cells as well as SKOV3 and OVCAR3 cells overexpressing YTHDC1 were subcutaneously injected into nude mice. 4-6 weeks later, YTHDC1 upregulation significantly suppressed the growth of the tumor as compared to the control group (Figure 3A-B and Figure S2F-G). Furthermore, the YTHDC1 overexpression enhanced the activity of caspase-3 but decreased the Ki-67 signaling in the tumor region as shown by IHC staining (Figure 3C-E). Altogether, these findings confirm the important function of YTHDC1 in inhibiting the progression of ovarian cancer both *in vitro* and *in vivo*.

### Identification of the YTHDC1 target candidates in ovarian cancer

Further investigation of the underlying molecular mechanism employed by YTHDC1 in the progression of ovarian cancer was performed by RNA-seq analysis in the SKOV3 and A2780 cells with YTHDC1 knockdown. As shown in Figure S4A, YTHDC1 knockdown in the two lines produced 106 differentially expressed genes (DEGs) in common, including 56 up-regulated genes and 50 down-regulated genes Figure S4A-B). KEGG enrichment analysis revealed AGE-RAGE signaling pathway, cholinergic synapse, and N-glycan biosynthesis, *etc*. as the top 20 enriched pathways (Figure S4C).

**Figure 4:**
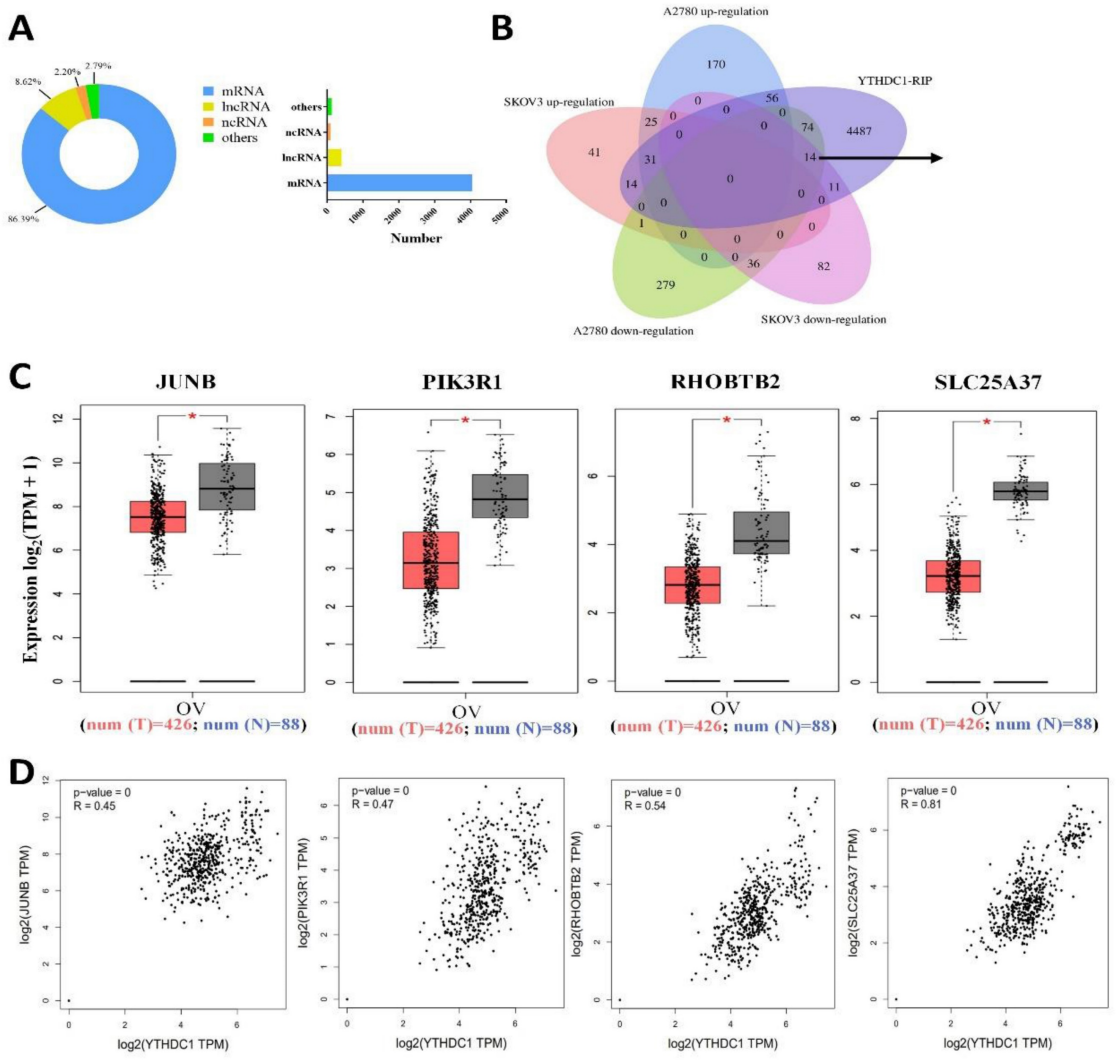
Identification of the YTHDC1 targets in ovarian cancer cells. (A) Distribution of RIP-seq identified YTHDC1 target transcripts. (B) Overlapping analysis of genes RIP-seq identified and genes described in Figure S4B). Fourteen common genes were screened out. (C and D) The expression of JUNB, PIK3R1, RHOBTB2, and SLC25A37 in normal tissues and ovarian cancer tissues using the GEPIA database (C), and their correlation with YTHDC1 expression (D).

Next, we applied YTHDC1-RIP-seq in SKOV3 cells to identify RNAs directly bound to YTHDC1. A total of 9522 potential targets of YTHDC1 were identified (Table S4, and 86.39% of them were mRNAs (Figure 4A). Recent studies have highlighted that YTHDC1 binds to m6A-modified mRNA and maintains its stability 20, 21, so YTHDC1 knockdown results in decreased expression of its target genes. Intriguingly, 14 genes overlapped in the RNA-seq (downregulated in SKOV3 and A2780) and RIP-seq data (Figure 4B). Furthermore, GEPIA database analysis showed that only four genes, PIK3R1, JUNB, RHOBTB2, and SLC25A37, were expressed at low level in ovarian cancer and were correlated positively with YTHDC1 (Figure 4C-D).

### PIK3R1 is the m6A modification target of YTHDC1

**Figure 5:**
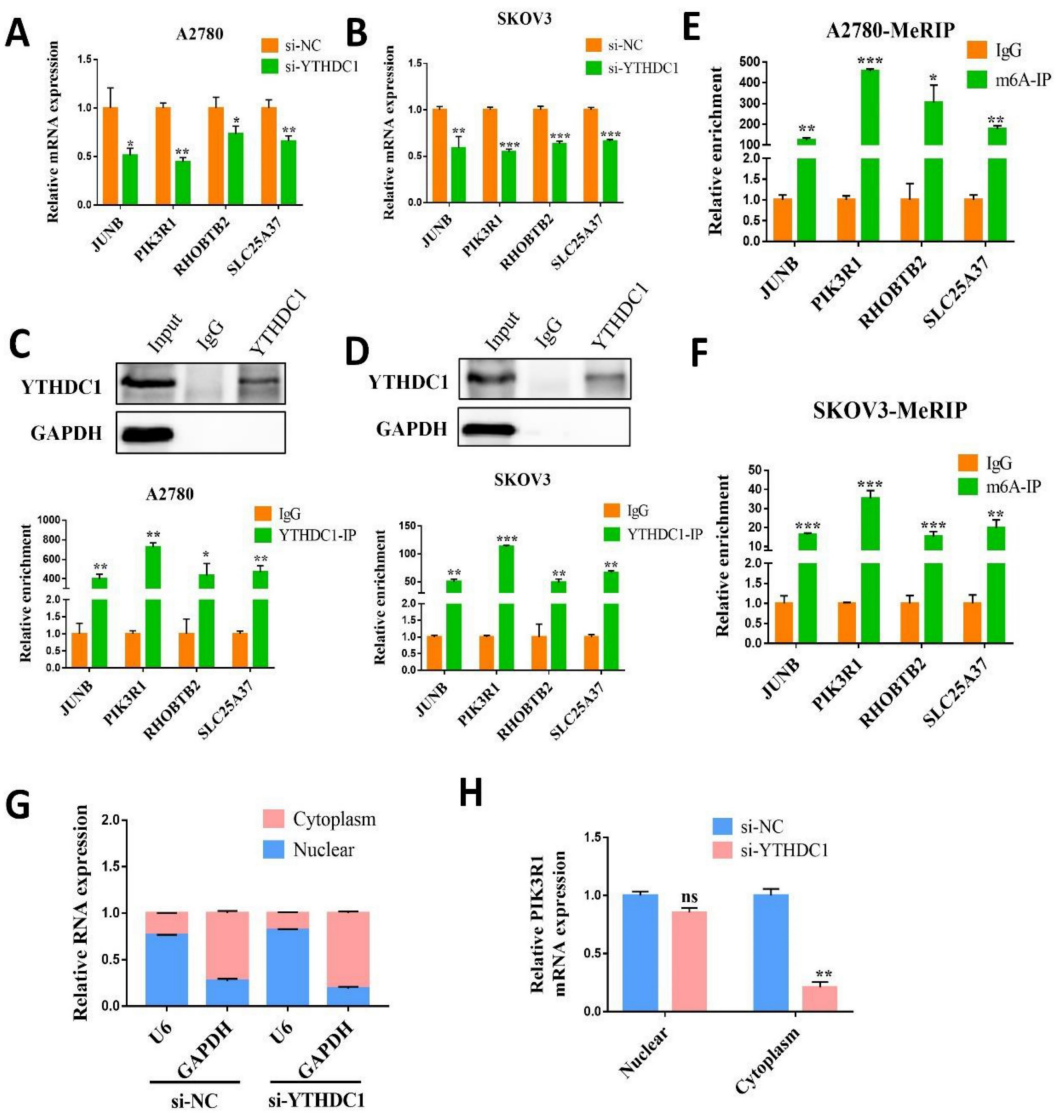
YTHDC1 targets PIK3R1 in ovarian cancer cells. (A and B) The mRNA level of JUNB, PIK3R1, RHOBTB2, and SLC25A37 in YTHDC1 knockdown A2780 (A) and SKOV3 (B) cells detected by qRT-PCR. (C and D) RIP-qPCR identified the interaction between YTHDC1 and the four target genes in A2780 (C) and SKOV3 (D) cells. (E and F) MeRIP-qPCR was performed to detect m6A levels of the four target genes in A2780 (E) and SKOV3 (F) cells. (G and H) The levels of PIK3R1 expression in cytoplasmic and nuclear of SKOV3 cell after YTHDC1 knockdown measured by qRT-PCR. GAPDH was used as a cytoplasmic marker and U6 as a nuclear marker. Data presented as means ± SD, *P < 0.05, **P < 0.01, ***P < 0.001.

**Figure 6:**
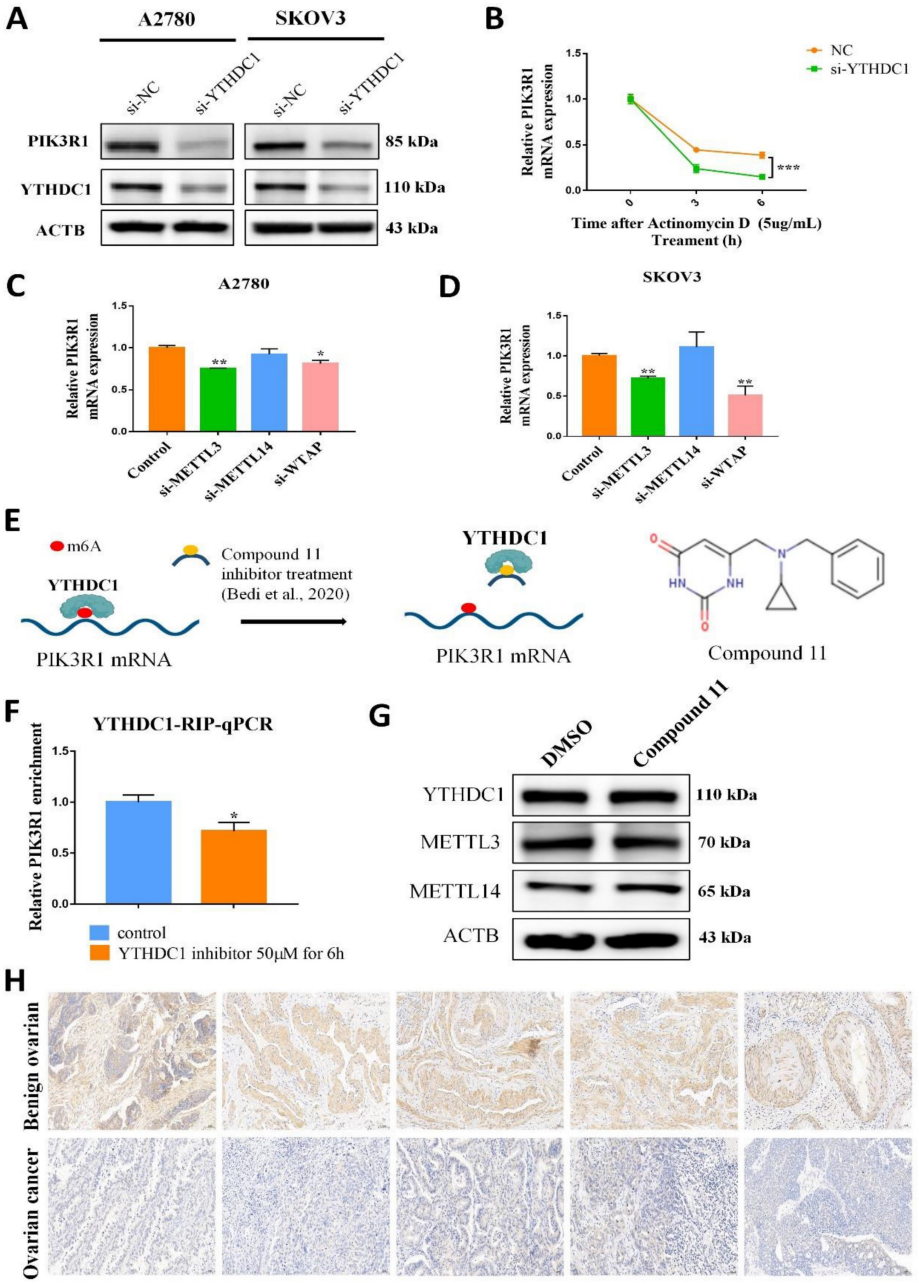
YTHDC1 recognizes and binds to PIK3R1 mRNA m6A site and maintains its stability. (A) The PIK3R1 protein level in SKOV3 and A2780 cells after YTHDC1 knockdown detected by western blotting. (B) The expression of PIK3R1 in YTHDC1 knockdown of A2780 cells after treatment with actinomycin D (5 µg/mL) detected by qRT-PCR. (C and D) The expression of PIK3R1 after knockdown of METTL3, METTL14, and WTAP respectively in A2780 (C) and SKOV3 (D) cells detected by qRT-PCR. (E) Schematic representation of perturbation of YTHDC1 and m6A-modified RNAs binding using an inhibitor. The chemical structure of compound 11 is shown (right). (F) RIP-qPCR detected the interaction between YTHDC1 and PIK3R1 in the A2780 cells after treatment with compound 11. (G) Indicated proteins and their expression was evaluated by western blot after 6 hours of YTHDC1 inhibitor treatment. (H) Representative immunohistochemistry results of PIK3R1 expression in cancerous and benign ovarian tissue. Data presented as means ± SD, *P < 0.05, **P < 0.01, ***P < 0.001.

To further investigate the targeted transcript of YTHDC1, we first validated the mRNA level of four aforementioned candidate genes in SKOV3 and A2780 cells with YTHDC1 knockdown. The results indicated that all four genes were reduced after YTHDC1 knockdown, among which PIK3R1 has the most significant downregulation (Figure 5A-B). As expected, the PIK3R1 protein level was significantly lowered following YTHDC1 knockdown (Figure 6A). Meanwhile, we found that PIK3R1 mRNA was more enriched by YTHDC1 protein using RIP-qPCR (Figure 5C-D). Additionally, we assessed the m6A modification levels of the four genes by MeRIP- qPCR and the highest m6A level was observed in PIK3R1 (Figure 5E-F). Together, we selected PIK3R1 as the target of YTHDC1 and performed further validation.

Interestingly, RNA nucleoplasmic separation showed PIK3R1 was significantly decreased in the cytoplasm, while there was no significant difference in the nucleus with YTHDC1 knockdown (Figure 5G-H). Therefore, we speculated that YTHDC1 might bind and stabilize m6A-modified PIK3R1 mRNA in the cytoplasm. Given that YTHDC1 positively regulated the mRNA level of PIK3R1, we then investigated whether YTHDC1 could affect the stability of PIK3R1. The SKOV3 and A2780 cells were treated with actinomycin-D at the indicated times and qRT-PCR revealed that YTHDC1 knockdown led to a significant decrease in PIK3R1 mRNA stability (Figure 6B). To figure out whether YTHDC1 binds to PIK3R1 through m6A modification, siRNAs of m6A methylases including METTL3, METTL14, and WTAP were designed and separately transfected into SKOV3 and A2780 cells. As expected, the expression of METTL3, METTL14, and WTAP was significantly decreased (Figure S5. As shown in Figure 6C-D, the PIK3R1 mRNA was markedly reduced after METTL3 and WTAP knockdown. In addition, compound 11, a chemical inhibitor, was recently reported to block the binding between YTHDC1 and m6A *in vitro* 23, 24 (Figure 6E). Results of the RIP-qPCR suggested that the binding of YTHDC1 to PIK3R1 mRNA was reduced by compound 11, but there was no alteration in the expression of YTHDC1 protein or m6A methylases (Figure 6F-G). Moreover, the binding of YTHDC1 to PIK3R1 mRNA was significantly decreased after knockdown of METTL3 Figure S6A-B). The PIK3R1 protein level in the ovarian cancer tissues were further assessed by IHC staining. Surprisingly, the PIK3R1 expression level in ovarian cancer tissues was markedly decreased compared to begin ovarian tissues (Figure 6H). Collectively, these results indicate that PIK3R1 is a direct target of YTHDC1 in ovarian cancer.

### Ectopic expression of PIK3R1 in ovarian cancer cells ameliorates the tumor-promoting impact of YTHDC1 deficiency

**Figure 7:**
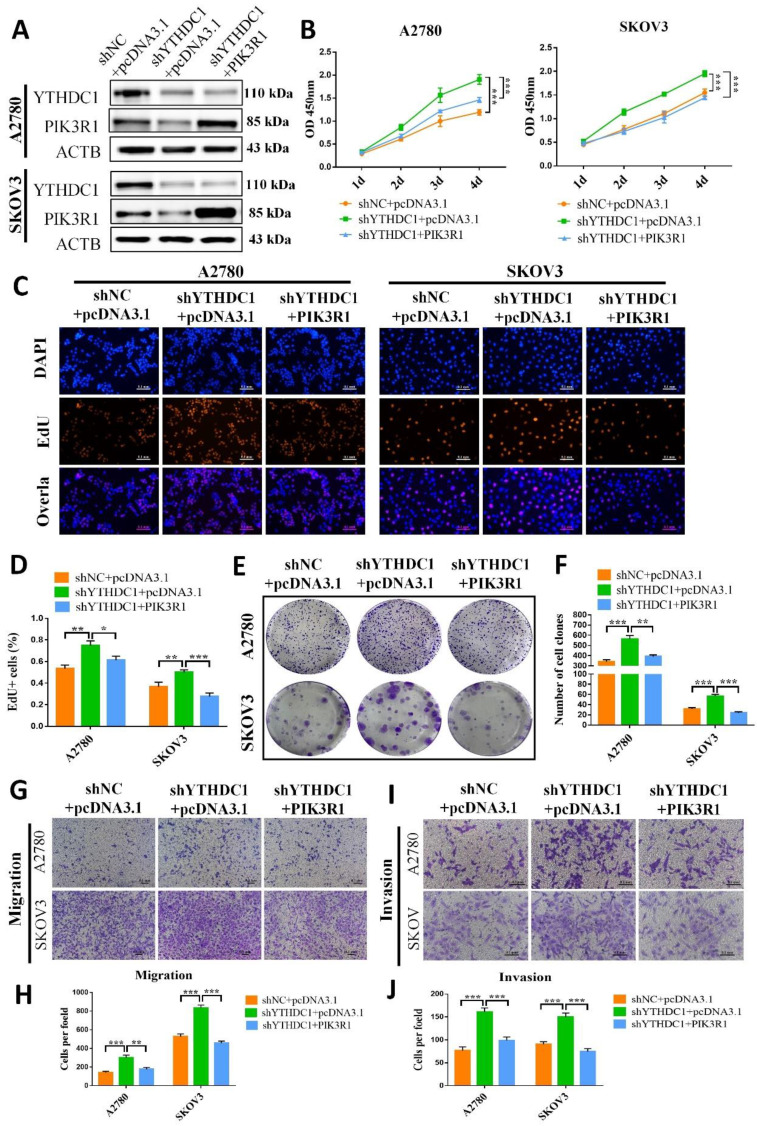
Low expression of YTHDC1 accelerates the malignant progression of ovarian cancer by downregulating PIK3R1. (A) The protein level of YTHDC1 and PIK3R1 in YTHDC1-deficient SKOV3 and A2780 cells after overexpression of PIK3R1 detected by western blotting. (B) CCK-8 assays to determine cell proliferation in SKOV3 and A2780 cells described in (A). (C and D) EdU assays for the detection of the proliferation rate of SKOV3 and A2780 cells mentioned in (A). Scale bar, 100 μm. (E and F) Colony formation assays to evaluate the SKOV3 and A2780 cell proliferation mentioned in (A). (G-J) Transwell assays for assessing the migration (G and H) and invasion (I and J) capacities of SKOV3 and A2780 cells described in (A). Data presented as means ± SD, *P < 0.05, **P < 0.01, ***P < 0.001.

The role of PIK3R1 in ovarian cancer was further explored by overexpressing PIK3R1 in YTHDC1 deficient SKOV3 and A2780 cells (Figure 7A). Results of CCK-8, EdU, and colony formation assays suggested that PIK3R1 overexpression could pose a reverse effect on the increased cell proliferation and colony-forming ability induced by YTHDC1 deficiency (Figure 7B-F). Consistently, the transwell assay revealed that cell migration and invasion were both promoted by YTHDC1 knockdown and were ameliorated following PIK3R1 overexpression (Figure 7G-J). Moreover, overexpression of PIK3R1 markedly suppressed shYTHDC1-induced ovarian cancer tumor growth *in vivo* (Figure S7. Thus, our data suggest that PIK3R1 as a crucial downstream target of YTHDC1 for inhibiting the progression of ovarian cancer.

### YTHDC1 deficiency enhances N-glycan biosynthesis in ovarian cancer through PIK3R1-activated expression of GANAB

**Figure 8:**
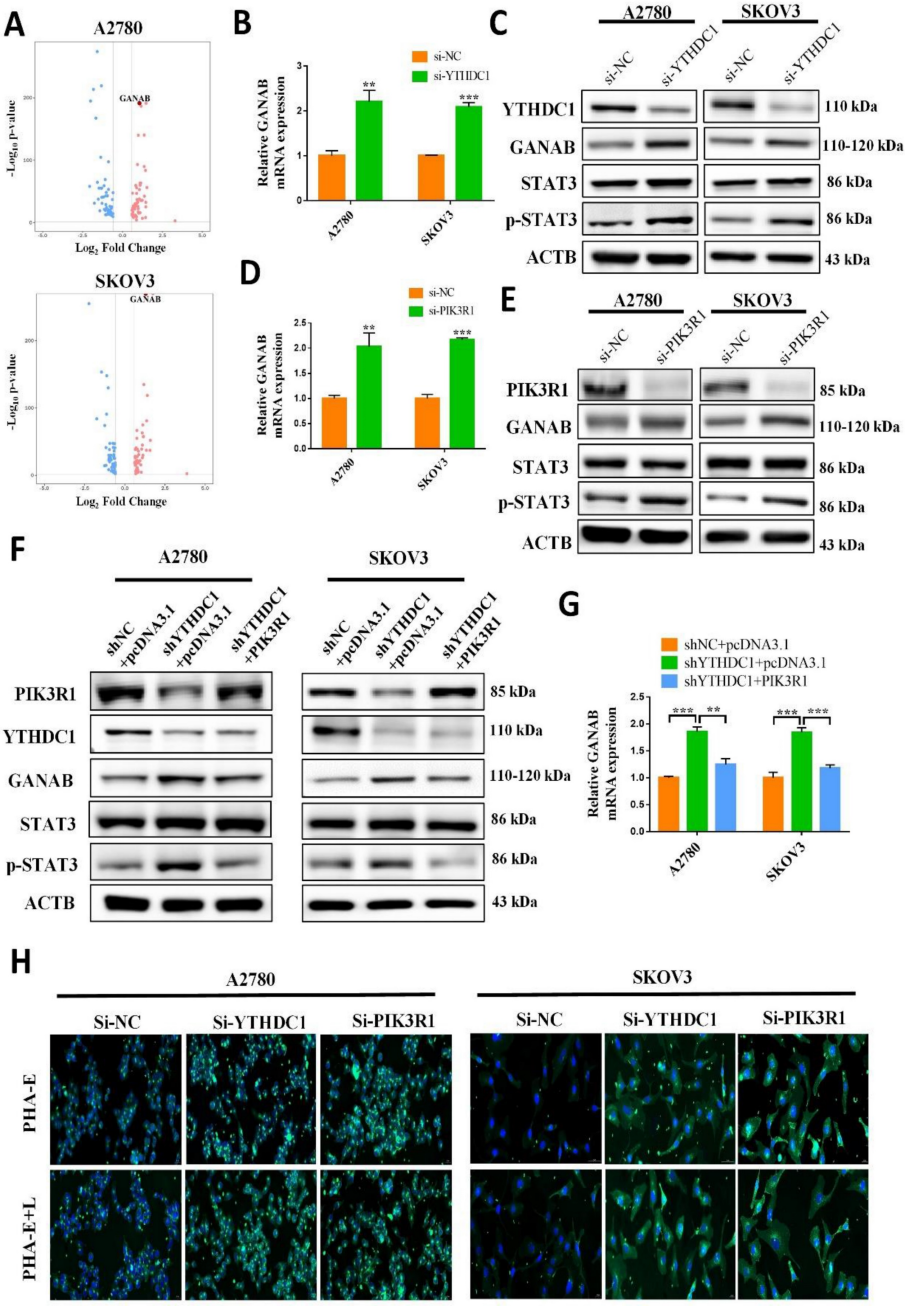
YTHDC1 deficiency enhances N-Glycan biosynthesis through the PIK3R1-p-STAT3-GANAB axis in ovarian cancer cells. (A) Volcano plot showing differentially expressed genes in SKOV3 and A2780 cells described in Figure S4B. Up-regulated genes and down-regulated genes are represented in red and blue, respectively. (B and C) Expression of PIK3R1, GANAB, STAT3, and p-STAT3 in SKOV3 and A2780 cells with YTHDC1 knockdown measured by qRT-PCR (B) and western blotting (C). (D and E) The expression of PIK3R1, GANAB, STAT3, and p-STAT3 in SKOV3 and A2780 cells with PIK3R1 knockdown measured by qRT-PCR (D) and western blot (E). (F and G) qRT-PCR (G) and western blot (F) detected the expression of PIK3R1, GANAB, STAT3, and p-STAT3 in YTHDC1-deficient SKOV3 and A2780 cells following PIK3R1 overexpression. (H) Bisecting GlcNAc and complex-type N-glycans expression profiles detected by PHA-E and PHA-E+L detected using lectin histochemistry in SKOV3 and A2780 cells with YTHDC1 and PIK3R1 knockdown, respectively. Scale bar, 20 μm. Data presented as means ± SD, *P < 0.05, **P < 0.01, ***P < 0.001.

RNA-seq data showed that metabolic pathway, especially N-glycan biosynthesis, was involved in ovarian cancer development with YTHDC1 knockdown (Figure 4C). Therefore, we examined whether YTHDC1 regulated N-glycan biosynthesis to affect ovarian cancer progression. The volcano plot showed that the expression of GANAB, which is associated with the N-glycan biosynthesis pathway, was markedly upregulated after YTHDC1 knockdown (Figure 8A). Then, qRT-PCR, as well as western blot analyses, revealed that although there was a significant increase in the expression of GANAB in SKOV3 and A2780 cells after YTHDC1 knockdown, these effects were reversed by PIK3R1 overexpression (Figure 8B-C and 8F-G). Furthermore, the protein and mRNA levels of GANAB underwent significant upregulation after PIK3R1 knockdown in SKOV3 and A2780 cells (Figure 8D-E), suggesting that GANAB is a downstream factor of PIK3R1. Considering that GANAB is a critical enzyme involved in N-glycan biosynthesis, we performed lectin fluorescence histochemistry in SKOV3 and A2780 cells. As shown in Figure 8H, after YTHDC1 or PIK3R1 knockdown, the expression level of complex-type N-glycans and bisecting GlcNAc recognized by PHA-E+L and PHA-E underwent an increase. These results suggested that YTHDC1 knockdown increased N-glycan biosynthesis by upregulating the expression of GANAB.

### YTHDC1 knockdown promotes the expression of GANAB through PIK3R1- STAT3 axis

**Figure 9:**
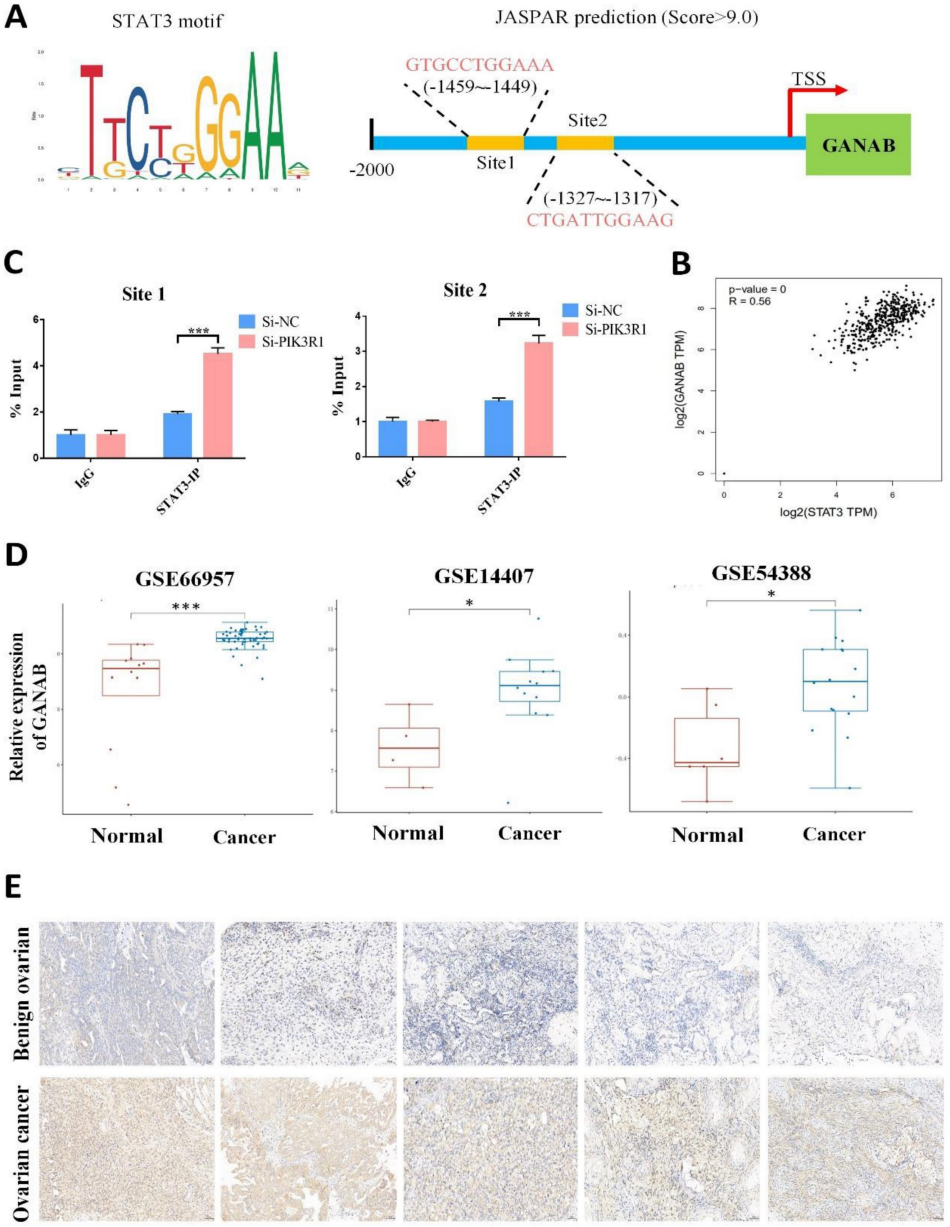
STAT3 transactivates the expression of GANAB by directly binding to its promoter. (A) Schematic illustration of potential sites located at the promoter region of GANAB for STAT3 binding as predicted by JASPAR. (B) ChIP-qPCR to detect STAT3 enrichment at GANAB promoter after PIK3R1 knockdown. (C) Search of the GEPIA database to examine the correlation between STAT3 and GANAB expression in ovarian cancer. (D) Relative level of GANAB RNA in cancer and normal ovarian tissues in GEO datasets. (E) Representative immunohistochemical images of expression level of GANAB in cancerous and benign ovarian tissues. Data presented as means ± SD, *P < 0.05, **P < 0.01, ***P < 0.001.

The potential mechanism by which PIK3R1 upregulates the expression of GANAB has been investigated. It has been reported that the loss of PIK3R1 could activate AKT and STAT3 signaling in ovarian cancer 25. Consistently, an increased STAT3 phosphorylation was observed in the SKOV3 and A2780 cells after PIK3R1 knockdown (Figure 8E). Moreover, we observed an increased level of p-STAT3 proteins after YTHDC1 knockdown, which was reversed by the overexpression of PIK3R1 (Figure 8C and 8F). As shown in Figure S8, the knockdown of YTHDC1 also resulted in translocation of STAT3 from the cytoplasm to the nucleus. STAT3 is regarded as a master transcription factor in the regulation of gene expression, thus we hypothesized that PIK3R1 promotes GANAB expression by activating STAT3 signaling. To address this question, we next used online database JASPAR (http://jaspar.genereg.net/) and found two consensus binding sites of STAT3 at the GANAB promoter region with score >9.0 (Figure 9A). To verify this, ChIP-qPCR was performed, and we found that STAT3 was highly enriched at the GANAB promoter after PIK3R1 knockdown (Figure 9B). Moreover, the GANAB protein level decreased after STAT3 knockdown, however, this effect was reversed by GANAB overexpression Figure S9A). Consistently, GANAB overexpression has the potential to reverse the reduced ovarian cancer cell proliferation, migration, and invasion ability induced by STAT3 deficiency (Figure S9B-F). Next, we investigated whether YTHDC1 knockdown effect could be affected by silencing GANAB. As a result, YTHDC1 knockdown significantly promoted ovarian cancer progression *in vivo* and* in vitro* and GANAB knockdown markedly suppressed ovarian cancer progression *in vivo* and* in vitro*, however, silencing GANAB abrogated shYTHDC1-induced tumor promotion (Figure S10A-F).

As expected, GEPIA database analysis revealed that there was a positive correlation between GANAB and STAT3 expression in ovarian cancer (Figure 9C). Furthermore, the GEO datasets confirmed that GANAB mRNA level were elevated in ovarian cancer (Figure 9D), which was also verified by IHC staining (Figure 9E). These findings suggested that PIK3R1 promoted GANAB expression by activating STAT3 signaling.

## Discussion

m6A, one kind of post-transcriptional modification first discovered in 1974(26, is believed to be the most common internal modification present in mRNAs 27, 28.

**Figure 10:**
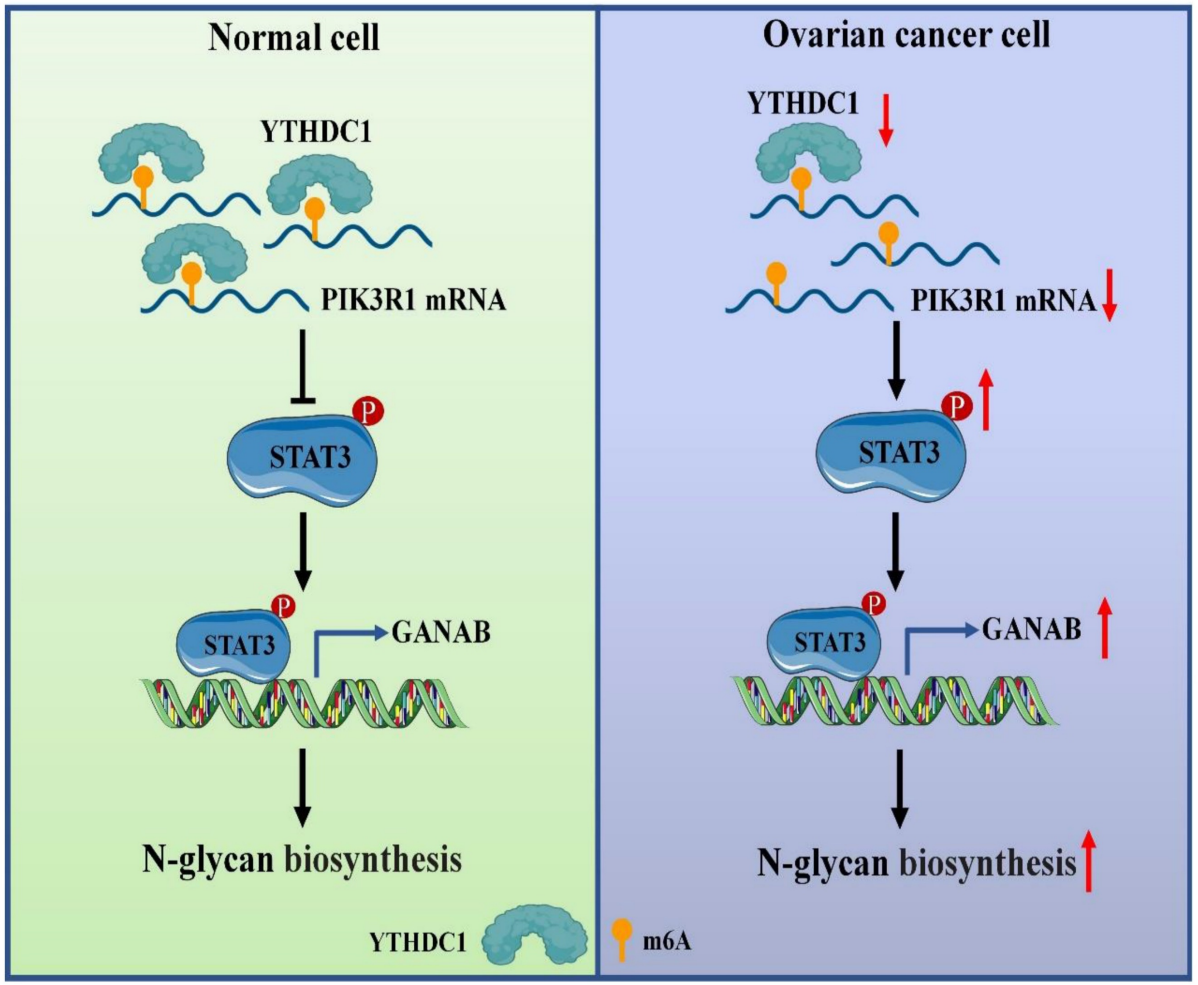
Proposed model underlying the roles of YTHDC1 in ovarian cancer

About 0.1-0.4% of adenines in mRNA undergo methylation at the N^6^ position, which represents 3-5 modifications on average per mRNA 29, 30. Furthermore, m6A exerts influence on post-transcriptional gene expression via the mechanism of altering the structure of RNA or specific recognition by m6A-binding proteins (called readers) during transcription 6, 31. Several studies have shown that the m6A modifications have a close association with carcinogenic or tumor suppressor capacity including tumorigenesis 32, proliferation 18, invasion 33, metastasis 34, 35 or immune system evasion 36 in malignant tumors. However, the regulation and function of m6A reader YTHDC1 in response to ovarian cancer are largely unknown. In this study, we demonstrated that YTHDC1 undergoes a significant reduction in its expression in ovarian cancer. Mechanistically, YTHDC1 recognizes the m6A sites on PIK3R1 mRNA, binds to it, and maintains the its stability. Moreover, a lower expression level of PIK3R1 induced by YTHDC1 knockdown leads to activation of STAT3 signaling, which further promotes GANAB expression in the nucleus, followed by an increase of GANAB-mediated N-glycan biosynthesis in ovarian cancer cells, which promotes ovarian cancer progression (Figure 10).

The recruitment of m6A-binding proteins is the primary mechanism via which m6A influences the destiny of mRNAs. The discovery of the first m6A-binding proteins, i.e., the YTH domain-containing proteins, established the foundation for understanding the function of m6A in mRNA 37. A total of five YTH domain-containing proteins have been described in the mammalian genome, including YTHDC1, YTHDC2, YTHDF1, YTHDF2, and YTHDF3, are the most important and noted readers 37-39. Among the m6A readers, YTHDF2 is the first reported reader protein to mediate the degradation of target mRNAs 40, YTHDF1 enhances the translation of m6A-modified mRNAs 41, and YTHDF3 and YTHDC2 have both functions 39, 42, 43. Furthermore, YTHDC1 has been linked to mRNA splicing 44, the nuclear export of mRNA 19, and mRNA stability 20, 21, 45 etc. Some recent studies have demonstrated that YTHDC1 plays important roles in several biological functions and disease progressions such as, embryonic development 46, neuronal development 47, lung cancer 48, leukemia 20, 21, colorectal carcinoma 49, etc. For instance, there is an overexpression of YTHDC1 in acute myeloid leukemia which helps in the maintenance of acute myelocytic leukemia cells proliferation and survival 20, 21. The abnormal expression of m6A-related regulatory enzymes including METTL3, FTO, ALKBH5, IGF2BP1, YTHDF1, and YTHDF2 is closely linked with the growth, metastasis, invasion, and chemotherapy resistance in ovarian cancer 50. However, the molecular mechanism by which YTHDC1 recognizes and regulates the expression of its target genes is still unknown in the development of ovarian cancer. Herein, we show that YTHDC1 has a low expression in ovarian cancer, suggesting that YTHDC1 may be a tumor suppressor gene. Consistently, our data demonstrated that YTHDC1 inhibits the growth and metastasis of ovarian tumors both *in vivo* and *in vitro*. Thus, YTHDC1 could be a prospective candidate for the development of a predictive biomarker and a promising target gene for the treatment of ovarian cancer.

To dissect YTHDC1's mechanisms involved in ovarian cancer, RIP-seq, RNA-seq, RIP-qPCR, and MeRIP-qPCR were conducted, and indicated that the key downstream target of YTHDC1 in ovarian cancer was PIK3R1. Previously, it was demonstrated that YTHDC1 maintains the m6A-modified mRNAs stability in the nucleus 21. Interestingly, our results indicated that YTHDC1 could bind and stabilize m6A-modified PIK3R1 mRNA mainly in the cytoplasm of the SKOV3 cell. PIK3R1, also known as p85α, is a well-known tumor-suppressor gene 51. Consistently, loss in copy numbers of PIK3R1 is commonly observed in several types of tumors such as prostate, ovarian, breast, and lung cancer 25, 52. Moreover, these tumor types have significantly lower level of PIK3R1 mRNA expression as compared to normal tissues. Emerging evidence has shown that the co-activation of AKT and STAT3 signaling pathways by PIK3R1 deletion enhances the progression of ovarian tumors 25. In the current study, we confirmed that PIK3R1 is significantly lowly expressed in ovarian cancer, thereby ameliorating the tumor-promoting property of YTHDC1 deficiency.

Subsequently, we found that GANAB gene expression, which is related to N-glycan biosynthesis, was remarkedly increased after YTHDC1 knockdown by RNA-seq analysis. Moreover, decreased PIK3R1 expression by YTHDC1 knockdown induces phosphorylation of STAT3, which further promotes its expression by binding to the promoter of GANAB in the nucleus. N-glycan is biosynthesized in the endoplasmic reticulum and the Golgi body. Furthermore, GANAB is involved in the cleavage of the last three glucose residues of the precursor oligosaccharide (GlcNAc2Man9Glc3), which works as a quality control step in monitoring protein folding in the endoplasmic reticulum 53, 54. Nevertheless, inhibition of GANAB results in N-glycoproteins that may be misfolded and excessively accumulate in the endoplasmic reticulum 55, and excessive endoplasmic reticulum stress leads to delayed cell proliferation and apoptosis 56. Several studies have shown that essential molecular and biological processes that occur in cancer, such as cell-cell communication, cell signaling pathways, tumor cell invasion, tumor angiogenesis, immune regulation, and metastasis, are all regulated by glycans 57, 58. In the tumor microenvironment, aberrant N-linked glycosylation is closely associated with cancer development, such as N-glycan biosynthesis inhibitors have anticancer activity in colorectal cancer 59. Herein, we have confirmed that the expression of GANAB is markedly increased in ovarian cancer tissues.

In summary, the expression of YTHDC1 was found to be downregulated in ovarian cancer. Our findings also demonstrate the role of YTHDC1 as a tumor suppressor in ovarian cancer development. Mechanistically, low expression of YTHDC1 promotes ovarian cancer tumorigenesis via PIK3R1/STAT3/GANAB axis. Therefore, our data may allow for a clear understanding of the pathogenesis of ovarian cancer and offer new insights into the diagnosis and treatment of ovarian cancer.

## Supplementary Material

Supplementary figures and tables 1-3.

Supplementary table 4.

## Abbreviations

YTHDC1: YTH Domain Containing 1
PIK3R1: Phosphoinositide-3-Kinase Regulatory Subunit 1
STAT3: Signal Transducer and Activator of Transcription 3
GANAB: Glucosidase II Alpha Subunit
FFPE: formalin-fixed paraffin-embedded
siRNA: small interfering RNA
qRT-PCR: quantitative real-time PCR
CCK-8: cell counting kit-8
EdU: 5-Ethynyl-2'-deoxyuridine
IHC: Immunohistochemistry
RNA-seq: RNA sequencing
RIP-seq: RNA immunoprecipitation and high-throughput sequencing
MeRIP: m6A-modified RNA immunoprecipitation
ChIP: Chromatin immunoprecipitation
DEGs: differentially expressed genes

## References

[1] Kuroki L, Guntupalli SR (2020). Treatment of epithelial ovarian cancer. BMJ (Clinical research ed).

[2] Matulonis UA, Sood AK, Fallowfield L, Howitt BE, Sehouli J, Karlan BY (2016). Ovarian cancer. Nature reviews Disease primers.

[3] Lisio MA, Fu L, Goyeneche A, Gao ZH, Telleria C (2019). High-Grade Serous Ovarian Cancer: Basic Sciences, Clinical and Therapeutic Standpoints. International journal of molecular sciences.

[4] Bitler BG, Watson ZL, Wheeler LJ, Behbakht K (2017). PARP inhibitors: Clinical utility and possibilities of overcoming resistance. Gynecologic oncology.

[5] Smith RA, Andrews KS, Brooks D, Fedewa SA, Manassaram-Baptiste D, Saslow D (2018). Cancer screening in the United States, 2018: A review of current American Cancer Society guidelines and current issues in cancer screening. CA: a cancer journal for clinicians.

[6] Huang H, Weng H, Chen J (2020). m(6)A Modification in Coding and Non-coding RNAs: Roles and Therapeutic Implications in Cancer. Cancer cell.

[7] Roundtree IA, Evans ME, Pan T, He C (2017). Dynamic RNA Modifications in Gene Expression Regulation. Cell.

[8] Meyer KD, Saletore Y, Zumbo P, Elemento O, Mason CE, Jaffrey SR (2012). Comprehensive analysis of mRNA methylation reveals enrichment in 3' UTRs and near stop codons. Cell.

[9] Tong J, Flavell RA, Li HB (2018). RNA m(6)A modification and its function in diseases. Frontiers of medicine.

[10] Batista PJ, Molinie B, Wang J, Qu K, Zhang J, Li L (2014). m(6)A RNA modification controls cell fate transition in mammalian embryonic stem cells. Cell stem cell.

[11] Zhao BS, Wang X, Beadell AV, Lu Z, Shi H, Kuuspalu A (2017). m(6)A-dependent maternal mRNA clearance facilitates zebrafish maternal-to-zygotic transition. Nature.

[12] Choe J, Lin S, Zhang W, Liu Q, Wang L, Ramirez-Moya J (2018). mRNA circularization by METTL3-eIF3h enhances translation and promotes oncogenesis. Nature.

[13] Liu J, Yue Y, Han D, Wang X, Fu Y, Zhang L (2014). A METTL3-METTL14 complex mediates mammalian nuclear RNA N6-adenosine methylation. Nature chemical biology.

[14] Zheng G, Dahl JA, Niu Y, Fedorcsak P, Huang CM, Li CJ (2013). ALKBH5 is a mammalian RNA demethylase that impacts RNA metabolism and mouse fertility. Molecular cell.

[15] Jia G, Fu Y, Zhao X, Dai Q, Zheng G, Yang Y (2011). N6-methyladenosine in nuclear RNA is a major substrate of the obesity-associated FTO. Nature chemical biology.

[16] Liao S, Sun H, Xu C (2018). YTH Domain: A Family of N(6)-methyladenosine (m(6)A) Readers. Genomics, proteomics & bioinformatics.

[17] Ma JZ, Yang F, Zhou CC, Liu F, Yuan JH, Wang F (2017). METTL14 suppresses the metastatic potential of hepatocellular carcinoma by modulating N(6) -methyladenosine-dependent primary MicroRNA processing. Hepatology (Baltimore, Md).

[18] Liu J, Eckert MA, Harada BT, Liu SM, Lu Z, Yu K (2018). m(6)A mRNA methylation regulates AKT activity to promote the proliferation and tumorigenicity of endometrial cancer. Nature cell biology.

[19] Roundtree IA, Luo GZ, Zhang Z, Wang X, Zhou T, Cui Y (2017). YTHDC1 mediates nuclear export of N(6)-methyladenosine methylated mRNAs. eLife.

[20] Cheng Y, Xie W, Pickering BF, Chu KL, Savino AM, Yang X (2021). N(6)-Methyladenosine on mRNA facilitates a phase-separated nuclear body that suppresses myeloid leukemic differentiation. Cancer cell.

[21] Sheng Y, Wei J, Yu F, Xu H, Yu C, Wu Q (2021). A critical role of nuclear m6A reader YTHDC1 in leukemogenesis by regulating MCM complex-mediated DNA replication. Blood.

[22] Qin Y, Zhong Y, Dang L, Zhu M, Yu H, Chen W (2012). Alteration of protein glycosylation in human hepatic stellate cells activated with transforming growth factor-β1. Journal of proteomics.

[23] Bedi RK, Huang D, Wiedmer L, Li Y, Dolbois A, Wojdyla JA (2020). Selectively Disrupting m(6)A-Dependent Protein-RNA Interactions with Fragments. ACS chemical biology.

[24] Lee JH, Wang R, Xiong F, Krakowiak J, Liao Z, Nguyen PT (2021). Enhancer RNA m6A methylation facilitates transcriptional condensate formation and gene activation. Molecular cell.

[25] Li X, Mak VCY, Zhou Y, Wang C, Wong ESY, Sharma R (2019). Deregulated Gab2 phosphorylation mediates aberrant AKT and STAT3 signaling upon PIK3R1 loss in ovarian cancer. Nature communications.

[26] Desrosiers R, Friderici K, Rottman F (1974). Identification of methylated nucleosides in messenger RNA from Novikoff hepatoma cells. Proceedings of the National Academy of Sciences of the United States of America.

[27] Yue Y, Liu J, He C (2015). RNA N6-methyladenosine methylation in post-transcriptional gene expression regulation. Genes & development.

[28] Nombela P, Miguel-López B, Blanco S (2021). The role of m(6)A, m(5)C and Ψ RNA modifications in cancer: Novel therapeutic opportunities. Molecular cancer.

[29] Chen M, Wei L, Law CT, Tsang FH, Shen J, Cheng CL (2018). RNA N6-methyladenosine methyltransferase-like 3 promotes liver cancer progression through YTHDF2-dependent posttranscriptional silencing of SOCS2. Hepatology (Baltimore, Md).

[30] Schibler U, Kelley DE, Perry RP (1977). Comparison of methylated sequences in messenger RNA and heterogeneous nuclear RNA from mouse L cells. Journal of molecular biology.

[31] Zaccara S, Ries RJ, Jaffrey SR (2019). Reading, writing and erasing mRNA methylation. Nature reviews Molecular cell biology.

[32] Liu S, Li Q, Chen K, Zhang Q, Li G, Zhuo L (2020). The emerging molecular mechanism of m(6)A modulators in tumorigenesis and cancer progression. Biomedicine & pharmacotherapy = Biomedecine & pharmacotherapie.

[33] Lin S, Choe J, Du P, Triboulet R, Gregory RI (2016). The m(6)A Methyltransferase METTL3 Promotes Translation in Human Cancer Cells. Molecular cell.

[34] Lin X, Chai G, Wu Y, Li J, Chen F, Liu J (2019). RNA m(6)A methylation regulates the epithelial mesenchymal transition of cancer cells and translation of Snail. Nature communications.

[35] Yue B, Song C, Yang L, Cui R, Cheng X, Zhang Z (2019). METTL3-mediated N6-methyladenosine modification is critical for epithelial-mesenchymal transition and metastasis of gastric cancer. Molecular cancer.

[36] Han D, Liu J, Chen C, Dong L, Liu Y, Chang R (2019). Anti-tumour immunity controlled through mRNA m(6)A methylation and YTHDF1 in dendritic cells. Nature.

[37] Dominissini D, Moshitch-Moshkovitz S, Schwartz S, Salmon-Divon M, Ungar L, Osenberg S (2012). Topology of the human and mouse m6A RNA methylomes revealed by m6A-seq. Nature.

[38] Xu C, Wang X, Liu K, Roundtree IA, Tempel W, Li Y (2014). Structural basis for selective binding of m6A RNA by the YTHDC1 YTH domain. Nature chemical biology.

[39] Hsu PJ, Zhu Y, Ma H, Guo Y, Shi X, Liu Y (2017). Ythdc2 is an N(6)-methyladenosine binding protein that regulates mammalian spermatogenesis. Cell research.

[40] Li J, Xie H, Ying Y, Chen H, Yan H, He L (2020). YTHDF2 mediates the mRNA degradation of the tumor suppressors to induce AKT phosphorylation in N6-methyladenosine-dependent way in prostate cancer. Molecular cancer.

[41] Wang X, Zhao BS, Roundtree IA, Lu Z, Han D, Ma H (2015). N(6)-methyladenosine Modulates Messenger RNA Translation Efficiency. Cell.

[42] Shi H, Wang X, Lu Z, Zhao BS, Ma H, Hsu PJ (2017). YTHDF3 facilitates translation and decay of N(6)-methyladenosine-modified RNA. Cell research.

[43] Kretschmer J, Rao H, Hackert P, Sloan KE, Höbartner C, Bohnsack MT (2018). The m(6)A reader protein YTHDC2 interacts with the small ribosomal subunit and the 5'-3' exoribonuclease XRN1. RNA (New York, NY).

[44] Xiao W, Adhikari S, Dahal U, Chen YS, Hao YJ, Sun BF (2016). Nuclear m(6)A Reader YTHDC1 Regulates mRNA Splicing. Molecular cell.

[45] Liang D, Lin WJ, Ren M, Qiu J, Yang C, Wang X (2022). m(6)A reader YTHDC1 modulates autophagy by targeting SQSTM1 in diabetic skin. Autophagy.

[46] Kasowitz SD, Ma J, Anderson SJ, Leu NA, Xu Y, Gregory BD (2018). Nuclear m6A reader YTHDC1 regulates alternative polyadenylation and splicing during mouse oocyte development. PLoS genetics.

[47] Shu L, Huang X, Cheng X, Li X (2021). Emerging Roles of N6-Methyladenosine Modification in Neurodevelopment and Neurodegeneration. Cells.

[48] Liu Z, Wang T, She Y, Wu K, Gu S, Li L (2021). N(6)-methyladenosine-modified circIGF2BP3 inhibits CD8(+) T-cell responses to facilitate tumor immune evasion by promoting the deubiquitination of PD-L1 in non-small cell lung cancer. Molecular cancer.

[49] Chen RX, Chen X, Xia LP, Zhang JX, Pan ZZ, Ma XD (2019). N(6)-methyladenosine modification of circNSUN2 facilitates cytoplasmic export and stabilizes HMGA2 to promote colorectal liver metastasis. Nature communications.

[50] Chang LL, Xu XQ, Liu XL, Guo QQ, Fan YN, He BX (2021). Emerging role of m6A methylation modification in ovarian cancer. Cancer cell international.

[51] Vallejo-Díaz J, Chagoyen M, Olazabal-Morán M, González-García A, Carrera AC (2019). The Opposing Roles of PIK3R1/p85α and PIK3R2/p85β in Cancer. Trends in cancer.

[52] Taniguchi CM, Winnay J, Kondo T, Bronson RT, Guimaraes AR, Alemán JO (2010). The phosphoinositide 3-kinase regulatory subunit p85alpha can exert tumor suppressor properties through negative regulation of growth factor signaling. Cancer research.

[53] Patterson MC (2005). Metabolic mimics: the disorders of N-linked glycosylation. Seminars in pediatric neurology.

[54] Qin Y, Zhao L, Wang X, Tong D, Hoover C, Wu F (2017). MeCP2 regulated glycogenes contribute to proliferation and apoptosis of gastric cancer cells. Glycobiology.

[55] Wu J, Ruas JL, Estall JL, Rasbach KA, Choi JH, Ye L (2011). The unfolded protein response mediates adaptation to exercise in skeletal muscle through a PGC-1α/ATF6α complex. Cell metabolism.

[56] Xu C, Bailly-Maitre B, Reed JC (2005). Endoplasmic reticulum stress: cell life and death decisions. The Journal of clinical investigation.

[57] Lau KS, Partridge EA, Grigorian A, Silvescu CI, Reinhold VN, Demetriou M (2007). Complex N-glycan number and degree of branching cooperate to regulate cell proliferation and differentiation. Cell.

[58] Zhao Y, Sato Y, Isaji T, Fukuda T, Matsumoto A, Miyoshi E (2008). Branched N-glycans regulate the biological functions of integrins and cadherins. The FEBS journal.

[59] de-Freitas-Junior JC, Bastos LG, Freire-Neto CA, Rocher BD, Abdelhay ES, Morgado-Díaz JA (2012). N-glycan biosynthesis inhibitors induce *in vitro* anticancer activity in colorectal cancer cells. Journal of cellular biochemistry.

