# Supplementary material for: Low expression of m6A reader YTHDC1 promotes progression of ovarian cancer via PIK3R1/STAT3/GANAB axis
Source: Int J Biol Sci. 2023 Aug 28;19(14):4672–88. doi: 10.7150/ijbs.81595 (PMC10535707; doi:10.7150/ijbs.81595)
Supplement: Supplementary file 1 — Supplementary figures and tables 1-3. [file ijbsv19p4672s1.pdf]

Table S1. SiRNA and shRNA used for silencing target genes.

Target transcript	Sequence (5'-3') sense	antisense
YTHDC1 siRNA	UGCCUCCAGAGAACCUUAUAA	UUAUAAGGUUCUCUGGAGGCA
METTL3 siRNA	GCCAAGGAACAAUCCAUUGUU	AACAAUGGAUUGUUCCUUGGC
METTL14 siRNA	CCAUGUACUUACAAGCCGAUA	UAUCGGCUUGUAAGUACAUGG
WTAP siRNA	AUGGCAAGAGAUGAGUUAAUU	AAUUAACUCAUCUCUUGCCAU
PIK3R1 siRNA	AGUAAAGCAUUGUGUCAUA	UAUGACACAAUGCUUUACU
STAT3 siRNA	GCACAAUCUACGAAGAAUCAA	UUGAUUCUUCGUAGAUUGUGC
YTHDC1 shRNA	TGCCTCCAGAGAACCTTATAATTATAAGGTTCTCTGGAGGCA	
GANAB shRNA	GCTGTGGATAGAAGCAACTTTAAAGTTGCTTCTATCCACAGC	

Table S2. Primers for qRT-PCR

Gene name	Forward primer 5'→3'	Reverse primer 5'→3'
β-actin	CATGTACGTTGCTATCCAGGC	CTCCTTAATGTCACGCACGAT
U6	GCTTCGGCAGCACATATACTAAAAT	CGCTTCACGAATTTGCGTGTCAT
GAPDH	ACAACTTTGGTATCGTGGAAGG	GCCATCACGCCACAGTTTC
YTHDC1	ATCTTCCGTTCGTGCTGTCC	GGACCATACACCCTTCGCTT
PIK3R1	TGGACGGCGAAGTAAAGCATT	AGTGTGACATTGAGGGAGTCG
JUNB	ACAAACTCCTGAAACCGAGCC	CGAGCCCTGACCAGAAAAGTA
RHOBTB2	CAGCCAGCTTTGACGTGTG	TTGCCCCGTAAGATCCCGT
SLC25A37	AGAAAATCATGCGGACCGAAG	TGGTGGTGGAAAACGTCATTTA
GANAB	AACATGACTCGGTTCAGGATTG	CATCACGACCAGAGACAGAAAG

Table S3. Primers used for ChIP assay.

Target promoter	Primer sequence (5'-3')	
	Forward	Reverse
GANAB-site1	TGTCCTCCACCGCAACATC	TCTCACTGCTCAACCTCCCT
GANAB-site2	CTCCGGCCCTGGGTAATATG	CCCTGGAGAAGTCCCGAATG

Figure S1

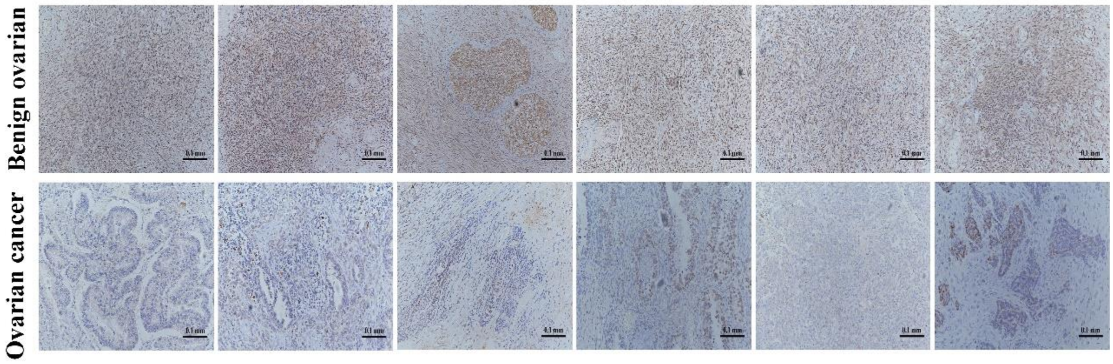

Figure S1. Representative immunohistochemical images of YTHDC1 expression in ovarian cancer tissue and benign ovarian tissue. Scale bar, 100 µm.

Figure S2

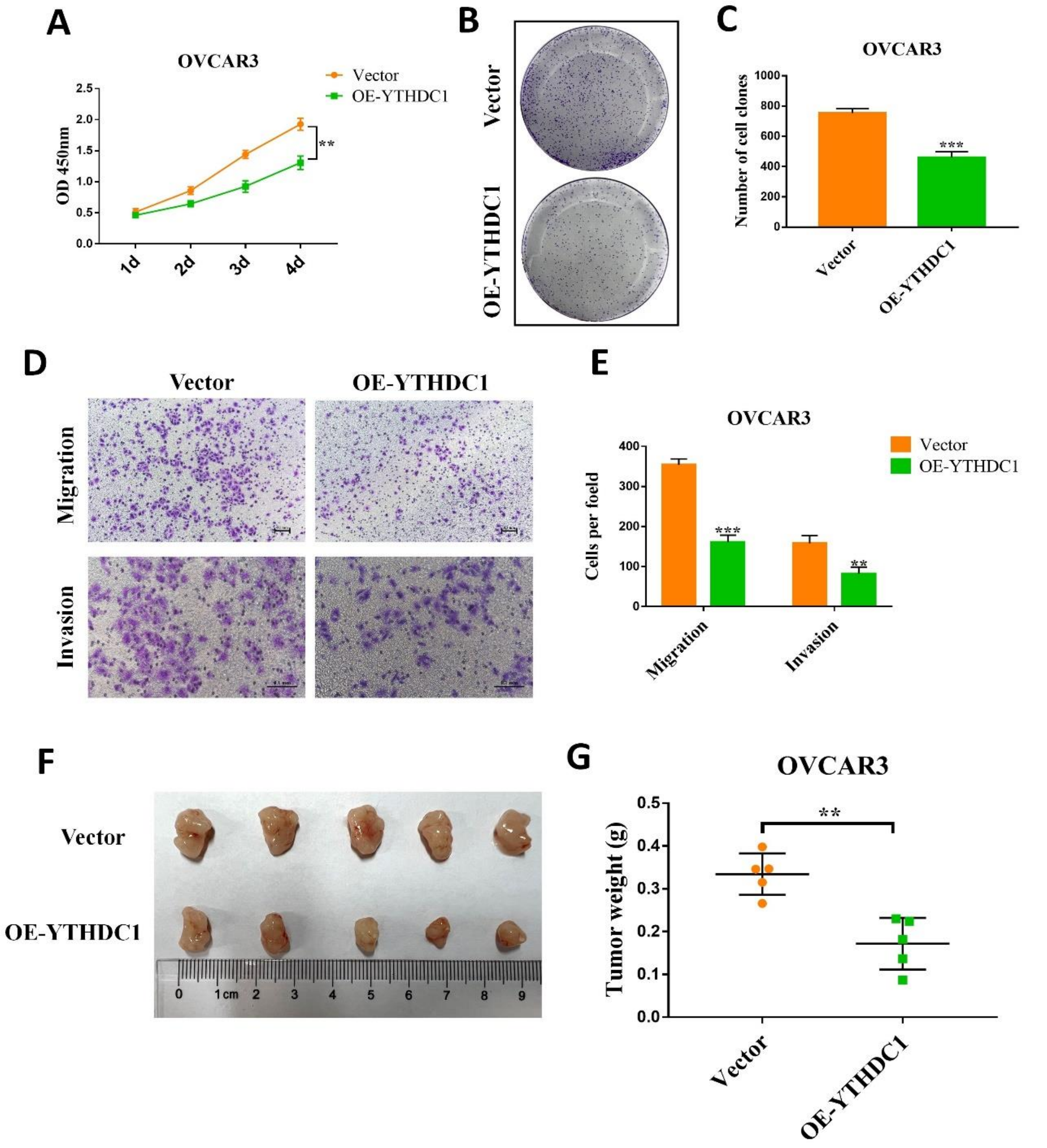

Figure S2. Overexpression of YTHDC1 inhibits the development of ovarian cancer. (A) CCK-8 assays were performed to determine cell proliferation after overexpression of YTHDC1 in OVCAR3 cell. (B and C) Colony formation assay of OVCAR3 cell was used to detect the proliferation. (D and E) Transwell assays were performed to detect cell migration and invasion using infected OVCAR3 cells. (F and G) OVCAR3 cells with empty vector or OE-YTHDC1 were subcutaneously injected into nude mice (n=5). The tumors were extracted and photographed at 28 days (F) after transplantation and the weight of tumors was measured (G). Data presented as means ± SD, **P*<0.05, ***P*<0.01, ****P*<0.001.

Figure S3

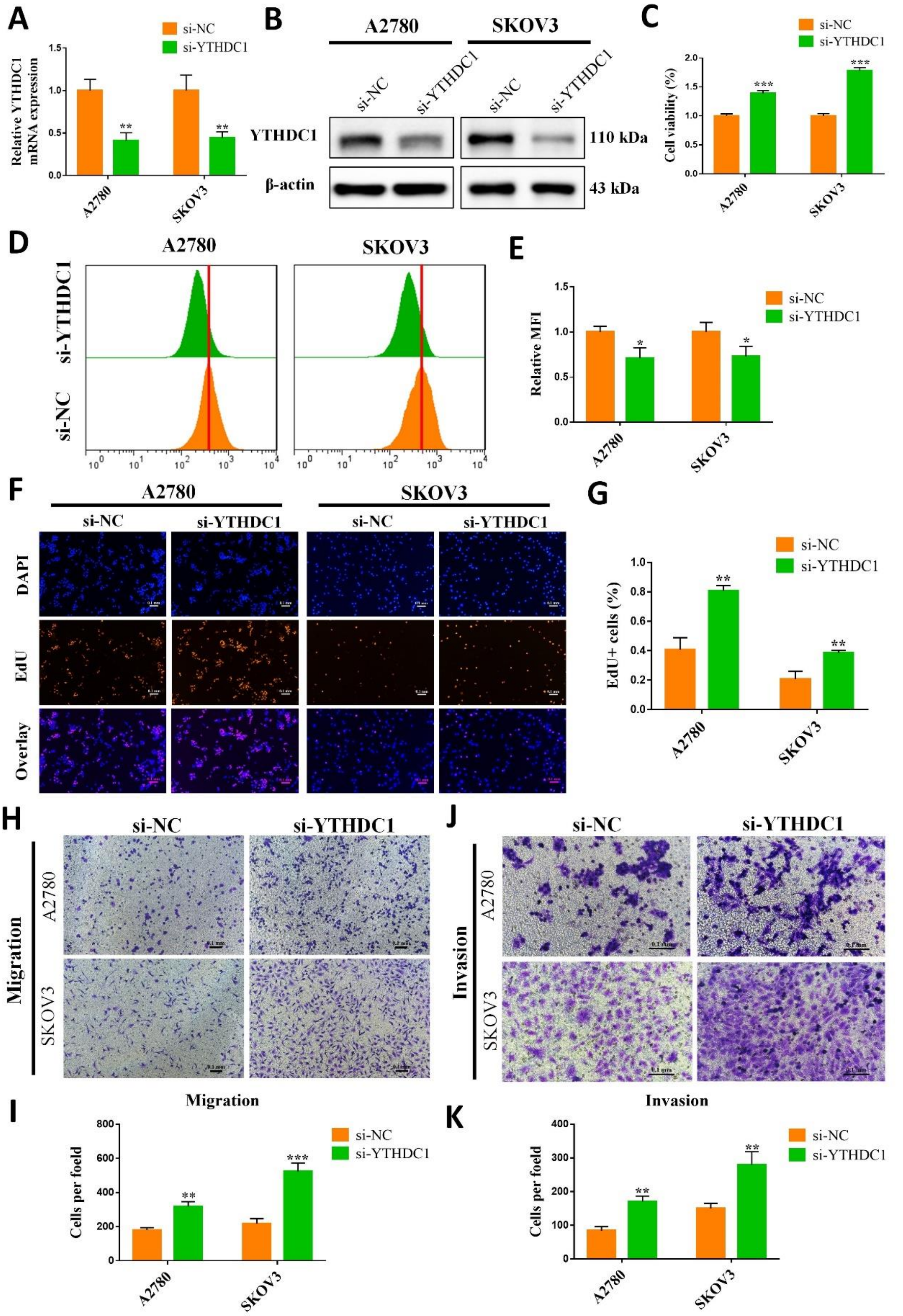

Figure S3. YTHDC1 deficiency induces development of ovarian cancer. (A and B) The levels of YTHDC1 expression in A2780 and SKOV3 cells after transfected with siRNA targeting YTHDC1 or a control siRNA were measured by qRT-PCR (A) and western

blot (B). (C) CCK-8 assays were performed to determine cell proliferation after knockdown of YTHDC1 in A2780 and SKOV3 cells. (D and E) Representative CFSE assay assessing the relative proliferation of A2780 and SKOV3 cells after knockdown of YTHDC1 for 36 h (D). Quantification data are shown as MFI (E) (n = 3). (F and G) EdU assays were used to detect the proliferation rate of A2780 and SKOV3 cells after transfection for 36 h. Scale bar, 100 μm. (H-K) Transwell assays were performed to detect cell migration (H and I) and invasion (J and K) using transfected A2780 and SKOV3 cells. Data presented as means ± SD, *$P < 0.05$, **$P < 0.01$, ***$P < 0.001$.

Figure S4

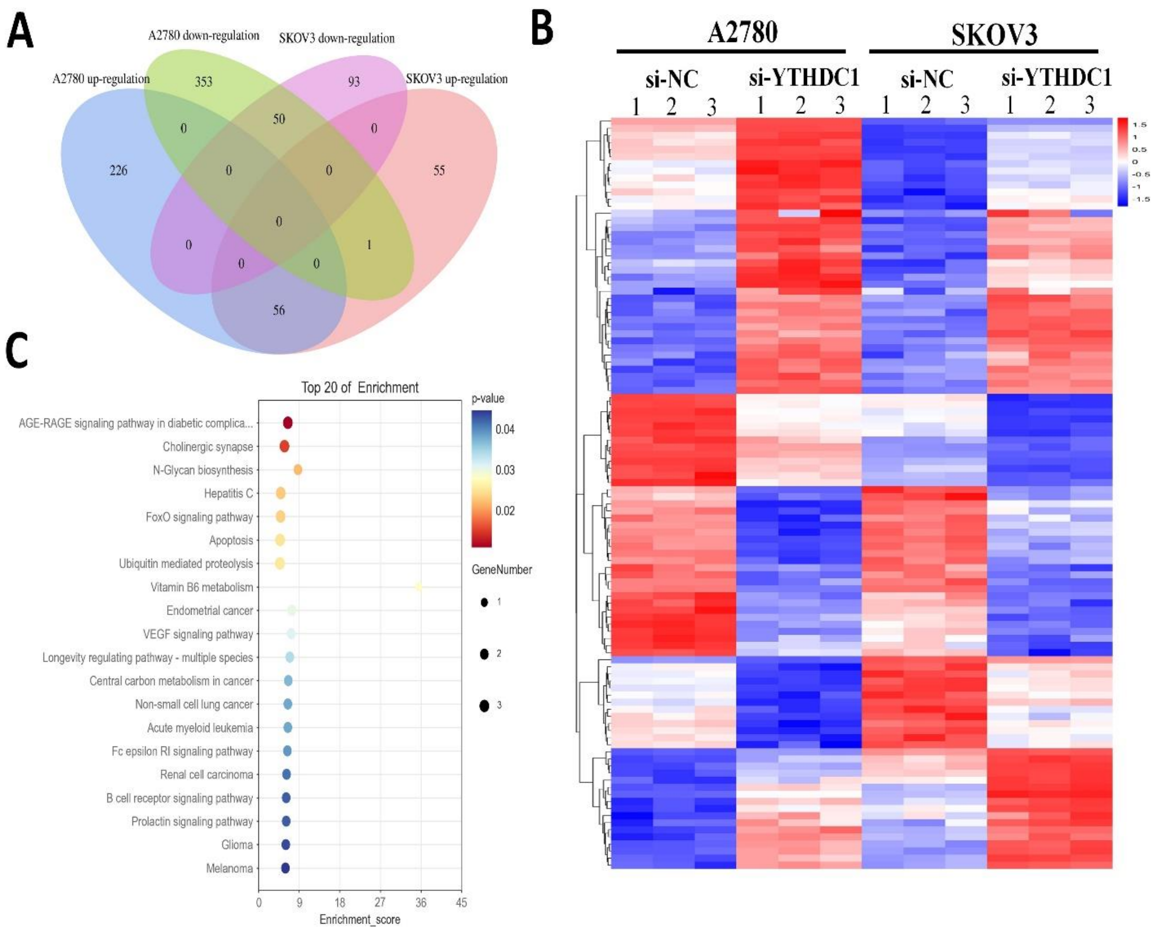

Figure S4. RNA-seq identified differentially expressed genes in YTHDC1 knockdown cells. (A) RNA-seq was performed after knockdown of YTHDC1 in A2780 and SKOV3 cells, and Venn diagram were plotted to show the intersection of differentially expressed genes in both two cells. (B) Heatmap was showed overlapping differentially expressed genes in two cells. (C) KEGG analysis of genes described in (B).

Figure S5

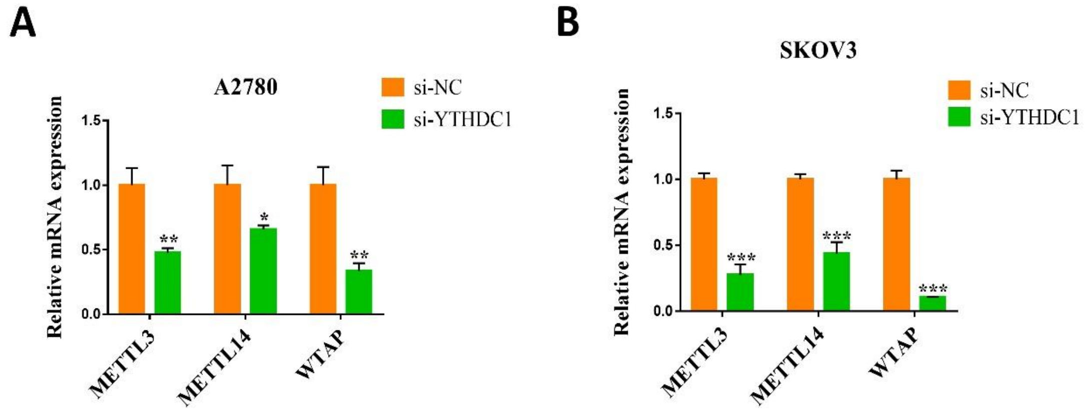

Figure S5. The expression of METTL3, METTL14, and WTAP in A2780 (A) and SKOV3 (B) cells were measured by qRT-PCR. Data presented as means ± SD, *P < 0.05, **P < 0.01, ***P < 0.001.

Figure S6

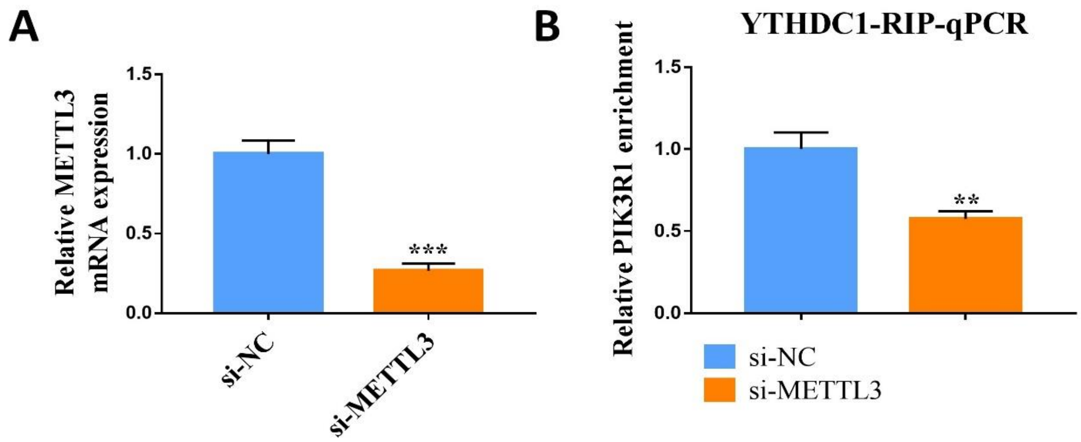

Figure S6. (A) The expression of METTL3 in SKOV3 cell was measured by qRT-PCR. (B) RIP-qPCR detected the interaction between YTHDC1 and PIK3R1 in SKOV3 cell after knockdown METTL3. Data presented as means ± SD, $^{**}P < 0.01$, $^{***}P < 0.001$.

Figure S7

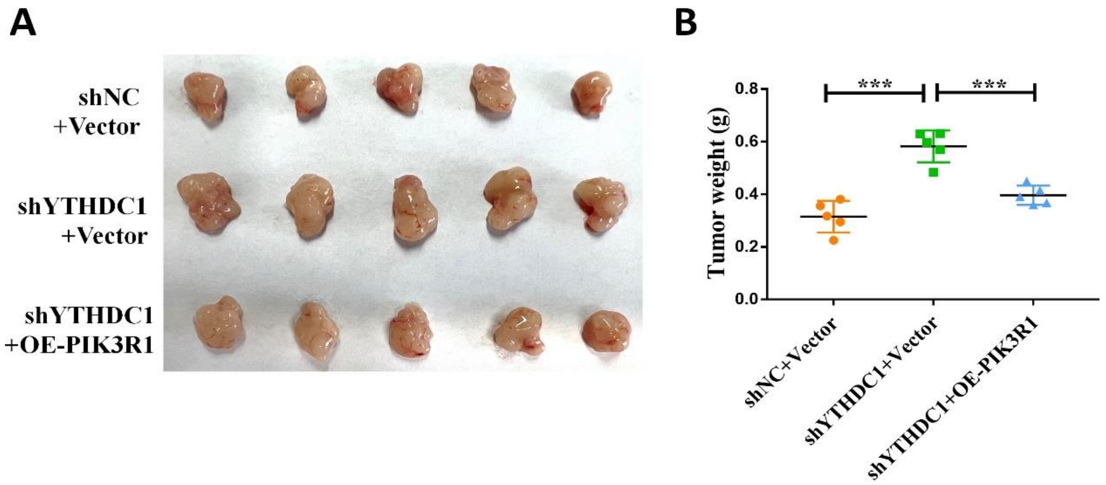

Figure S7. Overexpression of PIK3R1 ameliorates the tumor-promoting effect of YTHDC1 deficiency in vivo. (A and B) SKOV3 cells were stably infected with shYTHDC1, shYTHDC1 with OE-PIK3R1, or lentiviral vectors injected subcutaneously into five nude mice. The tumors were extracted, photographed (A) after 45 days transplantation and weighed (B).

Figure S8

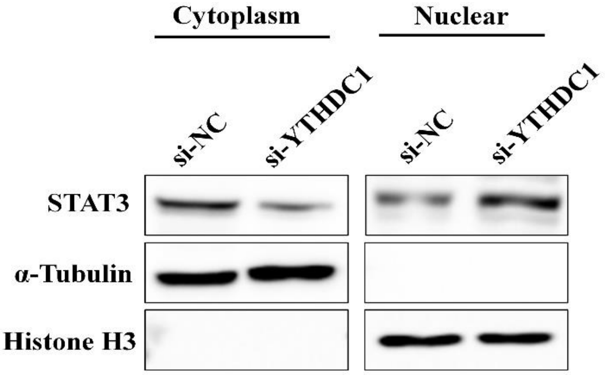

Figure S8. Detection of STAT3 expression levels in the cytoplasm and nucleus using western blot after knockdown of YTHDC1, α-Tubulin and Histone H3 were used as cytoplasm and nuclear markers, respectively.

Figure S9

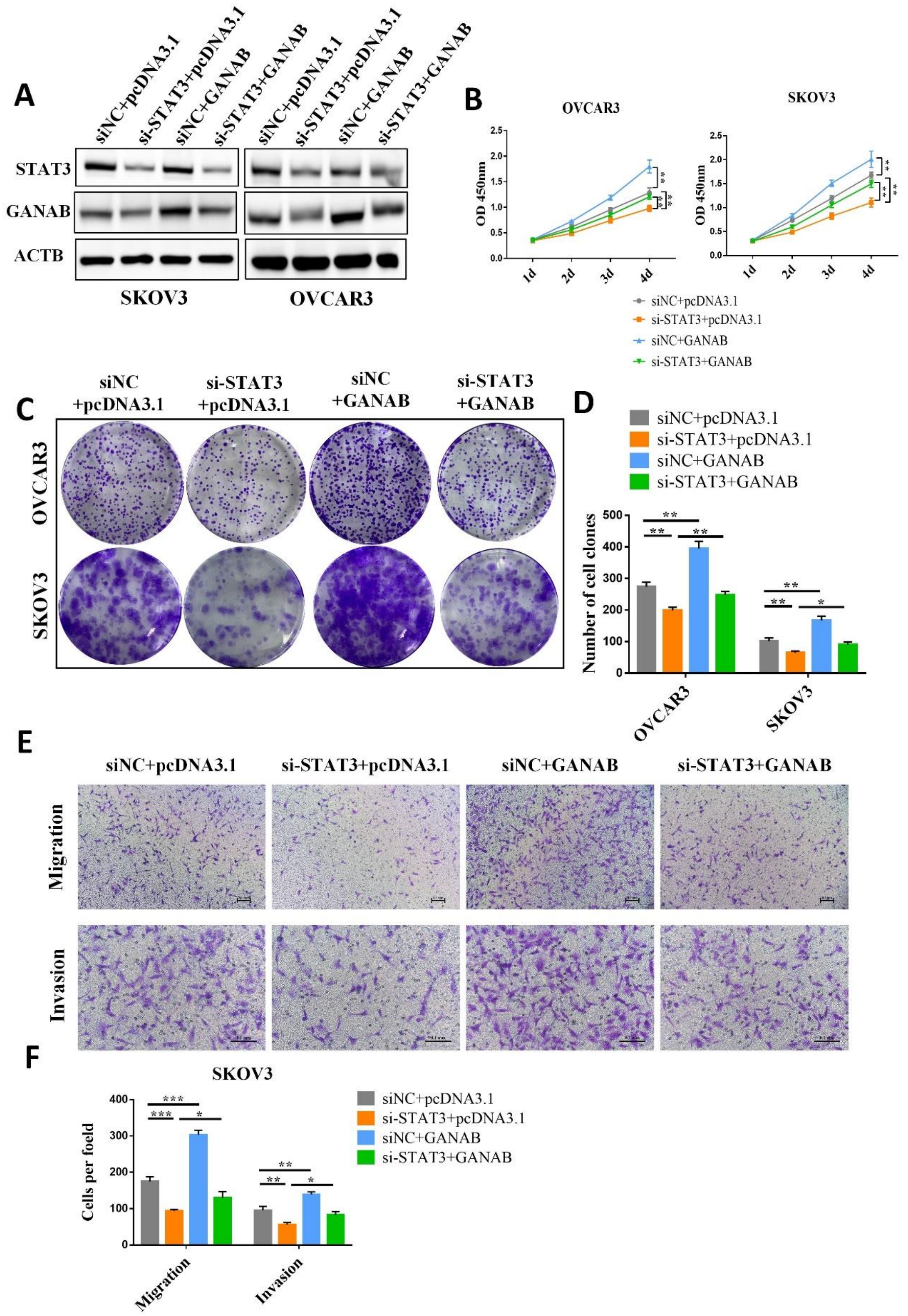

Figure S9. STAT3 knockdown inhibits malignant progression of ovarian cancer by downregulating GANAB. (A) The protein level of STAT3 and GANAB in STAT3-

deficient SKOV3 and OVCAR3 cells after overexpression of GANAB detected by western blotting. (B) CCK-8 assays to determine cell proliferation in SKOV3 and OVCAR3 cells described in (A). (C-D) Colony formation assays to evaluate the SKOV3 and OVCAR3 cells proliferation mentioned in (A). (E-F) Transwell assays for assessing the migration (E) and invasion (F) capacities of SKOV3 and OVCAR3 cells described in (A). Data presented as means ± SD, *P < 0.05, **P < 0.01, ***P < 0.001.

Figure S10

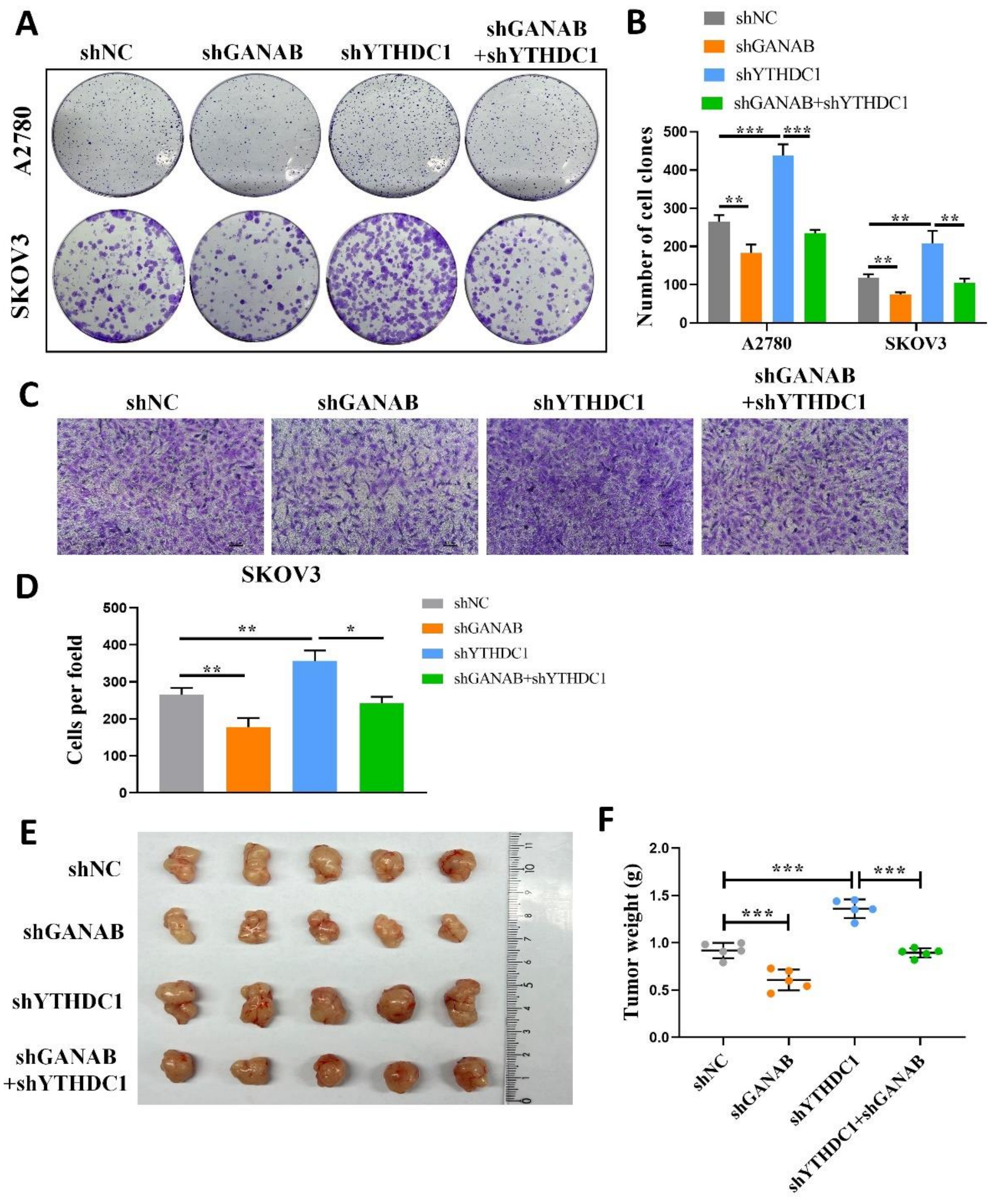

Figure S10. Knockdown of GANAB reversed shYTHDC1-induced tumor promotion *in vivo* and *in vitro*. (A and B) Colony formation assays to evaluate the A2780 and SKOV3 cells proliferation. (C and D) Transwell assays for assessing the migration of SKOV3 cell. (E and F) SKOV3 cells were stably infected with shYTHDC1, shGANAB, shGANAB with shYTHDC1, or shNC injected subcutaneously into five nude mice. The tumors were extracted, photographed (E) after 45 days transplantation and weighed (F).
